# Kinked Bisamides as Efficient Supramolecular Foam Cell Nucleating Agents for Low-Density Polystyrene Foams with Homogeneous Microcellular Morphology

**DOI:** 10.3390/polym13071094

**Published:** 2021-03-30

**Authors:** Bastian Klose, Daniel Kremer, Merve Aksit, Kasper P. van der Zwan, Klaus Kreger, Jürgen Senker, Volker Altstädt, Hans-Werner Schmidt

**Affiliations:** 1 Macromolecular Chemistry I, University of Bayreuth, Universitaetsstrasse 30, 95447 Bayreuth, Germany; bastian.klose@uni-bayreuth.de (B.K.); daniel.kremer@uni-bayreuth.de (D.K.); klaus.kreger@uni-bayreuth.de (K.K.); 2 Department of Polymer Engineering, University of Bayreuth, Universitaetsstrasse 30, 95447 Bayreuth, Germany; merve.aksit@uni-bayreuth.de; 3 Inorganic Chemistry III, University of Bayreuth, Universitaetsstrasse 30, 95447 Bayreuth, Germany; kasper.van-der-Zwan@uni-bayreuth.de; 4 Bavarian Polymer Institute and Bayreuth Institute of Macromolecular Research, University of Bayreuth, Universitaetsstrasse 30, 95447 Bayreuth, Germany

**Keywords:** supramolecular additives, foam nucleating agents, batch foaming, foam extrusion, cell nucleation, low-density polymer foams, microcellular foams, foam morphology

## Abstract

Polystyrene foams have become more and more important owing to their lightweight potential and their insulation properties. Progress in this field is expected to be realized by foams featuring a microcellular morphology. However, large-scale processing of low-density foams with a closed-cell structure and volume expansion ratio of larger than 10, exhibiting a homogenous morphology with a mean cell size of approximately 10 µm, remains challenging. Here, we report on a series of 4,4′-diphenylmethane substituted bisamides, which we refer to as kinked bisamides, acting as efficient supramolecular foam cell nucleating agents for polystyrene. Self-assembly experiments from solution showed that these bisamides form supramolecular fibrillary or ribbon-like nanoobjects. These kinked bisamides can be dissolved at elevated temperatures in a large concentration range, forming dispersed nano-objects upon cooling. Batch foaming experiments using 1.0 wt.% of a selected kinked bisamide revealed that the mean cell size can be as low as 3.5 µm. To demonstrate the applicability of kinked bisamides in a high-throughput continuous foam process, we performed foam extrusion. Using 0.5 wt.% of a kinked bisamide yielded polymer foams with a foam density of 71 kg/m^3^ and a homogeneous microcellular morphology with cell sizes of ≈10 µm, which is two orders of magnitude lower compared to the neat polystyrene reference foam with a comparable foam density.

## 1. Introduction

Polystyrene (PS) foams are widely used in a variety of applications, e.g., packaging, building and construction or in the automotive sector [1,2,3]. This is attributed to the low cost and established manufacturability of the polymer and foamed products, with the latter in particular excelling in their superior mechanical as well as thermal and acoustic insulation properties. These macroscopic properties are directly related to the foam morphology and foam density, with the key aspects being cell size and cell size distribution, cell density, and the open or closed cellular structure [1,4,5]. In this context, homogeneous microcellular and closed-cell foams with cell sizes of 10 µm or below as well as low foam densities with volume expansion ratios (VER) of larger than 10 are expected to show improved mechanical and thermal properties compared to macroscopic foams featuring cell sizes above 300 µm [1,2,4].

Numerous studies have been dedicated to investigating the structure–property relations of polymer materials and processing parameters in view of the foam cell morphology. This includes the influence of the physical blowing agent or mixture of those in view of the gas solubility and diffusivity and the rheological behavior of the (gas-loaded) polymer melt [3]. One important aspect is the use of additives and fillers, particularly to influence the melt strength or to act as heterogeneous nucleating agents for the foam cells to achieve polymer foams with a fine cellular or microcellular morphology [1,3]. For example, inorganic additives such as talc [6], silica [7,8] including mesoporous silica particle grafted with polystyrene brushes, (nano)clay [9,10], reduced and functional graphite oxide [11,12], expanded graphite [13], multi-walled carbon nanotubes [4,14,15], or carbon nanofibers [13,16] were investigated as additives. In general, inorganic additives tend to agglomerate within the polymer melt, reducing the effective surface-to-volume ratio, which together with a nonuniform distribution and dispersion of the additive leads to an inhomogeneous foam morphology [1,17].

An interesting aspect in this context is the use of shape-anisotropic inorganic additives, such as glass fibers with a high aspect ratio, which were beneficially employed to achieve foamed isotactic polypropylene (iPP) samples with water as a blowing agent. These injection-molded foamed specimens featured improved mechanical properties compared to the conventional injection molded ones [18]. Another interesting approach is based on the use of polymer fibers as additives to achieve fine cellular or microcellular foams, which was comprehensively investigated by the research group of Park [3]. In particular, they found that these fibrillated polymers improve the melt strength and foaming ability of thermoplastic polymers, such as polyethylene (PE) [19], iPP [20], or poly(lactic acid) (PLA) [21]. For instance, the addition of 5 wt.% of iPP nanofibers with diameters in the range of 100 nm to 300 nm to a PE melt resulted in a reduction of the average cell size from 900 µm to 28 µm. As a result, the cell density was significantly increased by four orders of magnitude. They also showed that adding 0.3–3.0 wt.% of polytetrafluoroethylene fibers (PTFE) with an average diameter of less than 500 nm and lengths above 100 µm to the thermoplastic polymer iPP improves the strain-hardening behavior of the melt, which leads to low-density foams with an increased cell density [20].

Apart from these insoluble additives based on inorganic materials or on polymer fibers used to improve the morphology of polymer foams, a rather novel class of additives is based on supramolecular materials. In contrast, the manner of functioning of supramolecular materials relies on the dissolution at elevated temperatures of the building blocks on a molecularly level in the polymer melt and the in situ formation of nano-objects via secondary interactions upon cooling the melt. Most typically, the formation and morphology of supramolecular objects depend on the molecular design of the building blocks, the polymer type, additive concentration, and other processing conditions such as temperature. Common examples, which are suitable to be used as additives in the polymer melt are based on naphthalene-bisamides [22], sorbitol-based derivatives [23] or benzenetrisamides (BTAs) [24]. In particular, BTAs have been successfully used as polymer crystal nucleating agents in various semi-crystalline polymers such as iPP [24,25], polybutylene terephthalate (PBT) [26], PLA [27], and polyvinylidene fluoride (PVDF) [28]. In a similar manner, such supramolecular nano-objects can be used as foam cell nucleating agents. Specifically, after the preparation of a polymer/additive mixture at ambient conditions, this approach involves dissolution of the supramolecular additive at elevated temperatures, resulting in homogeneously distributed building blocks in the polymer melt via molecular diffusion. Upon cooling, supramolecular nanoobjects are formed via secondary interactions by consumption of the dissolved building blocks, which effectively serves as heterogeneous nucleation sites for the added physical blowing agent. Since the homogeneously dispersed nano-objects provide a large number of predetermined nucleation sites for the physical blowing agent, this allows for control over the foam morphology with a homogeneous fine cellular structure (see the Supplementary Materials, Section S1). The potential as efficient foam nucleating agents has been successfully demonstrated by our research groups using selected BTAs for injection-molded iPP foams and extrusion foamed iPP, PS, and PBT [29,30,31,32,33]. However, using BTAs at large concentrations in nonpolar polymers remains challenging due to restrictions in the solubility and the concentration-dependent formation of larger sized objects. This probably limits the number of finely dispersed nano-objects in the polymer melt under conventionally employed conditions and ultimately restricts a very large number of nucleation sites necessary for a fine-celled polymer foam.

Recently, a novel class of supramolecular polymer additives, which were based on a 4,4′-diaminodiphenylmethane central unit with two side groups linked via two amide units, has been shown to efficiently act as crystal nucleating agents for iPP [34]. However, very little is known about this class of additives, which we refer to as kinked bisamides.

Here, we report on a comprehensive investigation of kinked bisamides with respect to structure–property relations regarding their bulk properties, self-assembly in solution, and their nano-object morphology as well as their capability to act as foam cell nucleating agents for polystyrene. The molecular design of these kinked bisamides was systematically varied at two selected positions (see Table 1) to tune the thermal properties and the self-assembly behavior of the supramolecular building blocks. Substitution at the ortho-positions of the amide groups with an increasing sterically demand of R_1_ from hydrogen to methyl or ethyl-groups enforces a torsion of the amide groups and consequently results in increased phase transitions of the kinked bisamides. Thus, we selected 4,4′-diaminodiphenylmethane, 4,4′-methylenebis-(2,6-dimethylaniline), and 4,4′-methylenebis(2,6-diethylaniline) to vary the structural features of the central unit. Similarly, systematical structural variations with respect to the peripheral side groups R_2_ were conducted using cyclohexane, phenyl, t-butyl, and n-butyl as peripheral side groups to investigate their thermal properties and the self-assembly behavior in the nonpolar solvent xylene. These two structural variations result in a total number of twelve kinked bisamides, which were utilized as foam cell nucleating agents in a thermally induced batch foam process of PS. A continuous foam extrusion process on technical scale was implemented to study the influence of a foam cell nucleating agent with respect to the foamability, foam morphology, and density of PS under harsh processing conditions including large shear forces, and high barrel temperatures and pressures. For both types of foaming processes, neat PS was prepared in the same manner to obtain a reference foam.

## 2. Materials and Methods

### 2.1. Materials

All solvents were purified and distilled according to standard procedures. The amines and acid chlorides employed were obtained from Sigma Aldrich (Darmstadt, Germany) and used as received.

The synthesis of all kinked bisamides based on selected 4,4′-diaminodiphenylmethane central units and aliphatic or aromatic side groups (Table 1) were performed via conversion of the respective amines with acid chlorides according to standard procedures. Details on the syntheses, purification and characterization are given in the Supplementary Materials, Section S2. Details on the thermal characterization and properties of all kinked bisamides are reported in the Supplementary Materials, Section S3.

PS 168N (INEOS Styrolution, Frankfurt am Main, Germany) was used as PS grade for batch and extrusion foaming experiments featuring a melt volume rate (MVR) (200 °C, 5 kg) of 1.5 cm^3^ (10 min)^−1^ and a bulk density ρ of 1040 kg·m^−3^ [35].

### 2.2. Self-Assembly of Kinked Bisamides and Nano-Object Characterization

For the self-assembly experiments to achieve and investigate supramolecular nano-objects in a nonpolar solvent at elevated temperatures, 500 ppm of the corresponding kinked bisamide was molecularly dissolved in 2 mL of xylene at 130 °C for 30 min in a closed vial. Subsequent cooling to room temperature yielded a turbid dispersion containing the self-assembled nanostructures. Prior to the morphology investigation via scanning electron microscopy (SEM), a drop of the suspension was deposited on a silicon wafer and the solvent was evaporated at ambient conditions. The samples were sputtered with a 2.0 nm thick platinum layer under argon atmosphere using a Sputter Coater 208HR (Cressington, Dortmund, Germany). SEM characterization of the samples were performed with a LEO Gemini 1530 FESEM (Zeiss, Jena, Germany) using an acceleration voltage of 2 to 3 kV. The dimensions of the supramolecular objects were measured with the program AxioVision (Zeiss, Jena, Germany). The reported data on the nano-objects’ dimensions refer to mean values of at least 70 determined objects for each sample.

### 2.3. Structural Studies of Kinked Bisamide ***3a***

To gain insight into the driving force for supramolecular nano-object formation and the corresponding morphology, structural methods were applied to reveal the crystal structure of kinked bisamide **3a**. Powder X-ray diffraction (PXRD) was performed on a crystalline powder of **3a** obtained from different solvents such as acetone and xylene. Prior to Rietveld refinement, density-functional theory (DFT) simulations were performed to optimize the molecular structure. Details on the structural elucidation are given in the Supplementary Materials, Section S4. Moreover, to provide additional evidence on the structural data, solid-state NMR (see the Supplementary Materials, Section S5) as well as Fourier-transform infrared spectroscopy (FTIR) on powder samples were performed (see the Supplementary Materials, Section S6).

### 2.4. Polymer Processing and Foaming

#### 2.4.1. Masterbatch Preparation

The polystyrene PS168N pellets were grinded in a ZM200 freezer mill (Retsch, Haan, Germany) using a mesh size of 1000 μm at a rotation speed of 18.000 rpm. The powdered PS was mixed with 1.0 wt.% (batch foaming) or 5.0 wt.% (extrusion foaming) of the finely powdered corresponding kinked bisamide. A Reax 2 overhead shaker (Heidolph, Schwabach, Germany) was used for 24 h at 50 rpm to achieve a homogeneous powder–powder mixture of the PS and kinked bisamides.

#### 2.4.2. Compounding and Preparation of Injection-Molded Specimen for Batch Foaming

Compounding was performed using a twin-screw micro-compounder, Xplore 15 mL (DSM, Heerlen, The Netherlands) under nitrogen atmosphere. As compounding conditions, a temperature of 260 °C, a residence time of 5 min, and a rotational speed of 50 rpm were selected. Different concentrations of 1.0, 0.75, 0.5, 0.25, 0.1, 0.05, 0.025, and 0.01 wt.% of the kinked bisamides in PS were achieved by preparing a dilution series. For this, 13.5 g of the masterbatch with a concentration of 1.0 wt.% of the kinked bisamide was compounded first. About 5.4 g of the molten polymer-additive mixture remained in the compounder after depletion, which was subsequently diluted to the selected concentration by adding a respective amount of neat PS granulates.

Injection molding was performed using a micro-injection-molding machine, Xplore 12 mL (DSM, Heerlen, The Netherlands). The barrel temperature of the injection molding unit was set to 250 °C, and an injection pressure of 6 bar was used. The molten compounded mixtures were transferred from the compounder to the micro-injection-molding machine and subsequently injected into a polished mold for the duration of 20s, yielding round platelets with a diameter of 27 mm and a thickness of 1.1 mm. To eliminate the internal stress of the specimens resulting from the injection molding process, the samples were annealed in a closed iron mold at 135 °C for 4 h.

#### 2.4.3. Batch Foaming

To saturate the injection-molded specimens with a physical blowing agent, a HR 500 high-pressure autoclave (Berghof, Eningen, Germany) was used. Saturation was performed at room temperature using CO_2_ at a pressure of 50 bar for 24 h. A CO_2_ uptake of approximately 6.5 wt.% was determined by weighing the specimens prior foaming. Batch foaming experiments were performed by immersing the CO_2_-loaded injection-molded specimens in a silicon oil bath at 130 °C for 15 s. The foamed samples were allowed to cool in an oil bath at ambient conditions for 20 s and then in water bath for a further 20 s. Oil residues were removed by washing the foams with soap water, and the foamed specimens were dried at ambient conditions for 24 h prior analysis.

#### 2.4.4. Extrusion Foaming

Foam extrusion experiments were carried out using a tandem extrusion line (Dr. Collin GmbH, Maitenbeth, Germany) comprising a twin-screw extruder with a 25 mm screw and a L/D ratio of 42 (A-Extruder), and a single-screw extruder with a 45 mm screw and L/D ratio of 30 (B-Extruder) equipped with a slit die with a gap of 0.6 mm and a width of 30 mm. Extruded PS (XPS) foams with the kinked bisamide **3a** at three selected concentrations, i.e., 0.1, 0.2, and 0.5 wt.%, were produced. For this, a masterbatch powder–powder mixture with 5.0 wt.% of **3a** was prepared in the same manner as described above. XPS foams with varying concentrations of **3a** were obtained by diluting the masterbatch with neat PS granulates by controlling the flow rates. A combination of 4 wt.% CO_2_ and 3 wt.% EtOH was used as a physical blowing agent. This ratio was found to be highly beneficial, since this composition can act as an efficient plasticizer for the polymer melt resulting in a homogeneous foam morphology [11,30]. As processing parameters, a screw speed of 8 rpm at the B-Extruder with an overall throughput of 4.5 kg h^−1^ was selected. The melt temperature in the A-Extruder was adjusted to 220 °C to ensure complete dissolution of the kinked bisamide **3a** in the PS melt. The melt temperature near the outlet of the B-extruder and the die temperature were selected between 110–120 °C and 126 °C, respectively. PS reference foams were prepared using neat PS granulates in the same manner.

### 2.5. Foam Characterization

#### 2.5.1. Foam Morphology

Batch or extrusion foam morphologies, which were evaluated with respect to the cell sizes and their distribution, were investigated via SEM imaging. Prior to the SEM investigation, the foamed specimens were cryo-fractured under liquid nitrogen yielding uniform breaking edges. The cryo-fractured specimens were clued on a silicon wafer and additionally wrapped with a copper foil to ensure better conductivity. The samples were sputtered with a 2-nm thick platinum layer under argon atmosphere using a Sputter Coater 208HR (Cressington). The field emission-scanning electron microscope, LEO Gemini 1530 FESEM (Zeiss), with an acceleration voltage of 3 kV was used to investigate the foam morphologies. The areas of the foam cells A_cell_ were determined using the software ImageJ. Assuming that the cells are circular, the cell diameters D_cell_ were calculated according to following equation:

(1) Dcell = 2Acellπ

The areas and corresponding cell diameters of at least 70 cells of each foam sample were taken into account, and the average values were given.

#### 2.5.2. Foam Density

Foam densities were calculated using the water-displacement method in agreement with ISO 1183 based on Archimedes’ principle using a XP 205 balance with density kit (Mettler Toledo). Samples were prepared by cutting a small rectangle of the foamed specimen, which was subsequently weighed in air (m_air_). The cut specimen was submerged in water, and its apparent mass (m_water_), which was reduced by the buoyant force, was measured. The foam density (ρ_foam_) was given according to the following equation [4].

(2) $$\rho_{foam}\;=\;\frac{m_{air}}{m_{air}-m_{water}}\;\cdot \;\rho_{water}\;\mathrm{with}\;\rho_{\mathrm{water}}\;(296.3\;K)\;=\;0.997541\;g/{\mathrm{cm}}^{3}\;$$

Foam densities were measured on at least three cut samples from different locations of the foam, and the mean values were given.

#### 2.5.3. Cell Density

Cell densities ρ_cell_ with respect to the unfoamed solid polymer were calculated according to Equation (3), with N_c_ being the number of cells in the selected area and A_S_ being the area of the selected section [4,36,37].

(3) $$\rho_{cell}={\left(\frac{N_{c}}{A_{s}}\right)}^{\frac{3}{2}}\cdot VER\;$$

with the volume expansion ratio (VER) calculated by dividing the average foam density by the polystyrene density, according to Equation (4).

(4) $$VER\;=\;\frac{\rho_{PS,bulk}}{\rho_{foam}}\;\;\mathrm{with}\;\rho_{\mathrm{PS},\mathrm{bulk}\;}=\;1040\;\mathrm{kg}/m^{3}\;$$

## 3. Results and Discussion

### 3.1. Synthesis, Characterization, and Thermal Properties of Kinked Bisamides

The class of supramolecular building blocks based on a 4,4′-diaminodiphenylmethane central unit with two peripheral side groups linked via two amide units, which we refer to here as kinked bisamides (see Table 1), is rather unnoticed and less investigated, in particular, in view of their thermal properties, self-assembly capabilities, and their use as foam cell nucleating agents. To reveal the structure–property relationships of this class, we systematically designed and synthesized a total of twelve kinked bisamides. In general, we anticipated that the central methylene group results in a kinked structure, which improves the solubility in nonpolar media. Structural variations of the molecular design were performed with respect to the substitution pattern of the ortho-position to the amide units (R_1_) as well as to the peripheral side groups (R_2_). Variation in the substituents R_1_ enforces a torsion of the amide groups due to an increasing steric demand from hydrogen to the methyl or ethyl groups, influencing the melting behavior of the kinked bisamides. Thus, we selected 4,4′-diaminodiphenylmethane, 4,4′-methylenebis-(2,6-dimethylaniline), and 4,4′-methylenebis(2,6-diethylaniline) as central unit molecules. Systematical structural variations in the peripheral side groups to further tune the thermal properties were conducted with respect to bulkiness and stiffness of the side groups R_2_ using the bulkier cyclohexane and phenyl side groups as well as the smaller but stiff t-butyl and the more flexible n-butyl side groups.

Syntheses of the kinked bisamides were performed according to standard procedures in a straightforward manner, converting the amine moieties of the central units with the corresponding acid chlorides of the peripheral side groups. To ensure sufficient solubility of the products, tetrahydrofurane (THF) or N-methyl-2-pyrrolidone (NMP) was used as a solvent and a tertiary amine such as triethylamine or pyridine was used as a hydrogen chloride scavenger. All kinked bisamides were obtained in high yield, ranging from 56 to 95%, and were identified and characterized by common analytical methods of the organic chemistry. The purity of the compounds was determined by means of HPLC and found for most of the compounds to be more than 95%. Details on the syntheses, purification, and characterization are given in the Supplementary Materials, Section S2. The thermal stabilities and the phase transition temperatures of all compounds were determined by thermogravimetric analysis (TGA) and differential scanning calorimetry (DSC) experiments (see the Supplementary Materials, Section S3). The T_-5wt.%_ values were close to 300 °C or higher, demonstrating a high thermal stability of the compounds. With the exception of **3d**, the melting temperatures were in the range of 190 °C to 320 °C and the crystallization temperatures were in the range of 170 °C to 310 °C. These findings suggest that these kinked bisamides can be processed at high temperatures and can self-assemble at elevated temperatures in the polymer melt. Both are a prerequisite for the additives to be used as supramolecular foam cell nucleating agents.

### 3.2. Self-Assembly of Kinked Bisamides from Xylene

An important aspect is the morphology of the supramolecular objects, which may act as nucleating sites for the cell formation. To gain insight on how the morphology and shape of the supramolecular objects may develop in a nonpolar polymer melt, self-assembly experiments were performed at elevated temperatures using a nonpolar solvent because solvents allow for straightforward isolation of the objects and their subsequent investigation by microscopic techniques. Xylene was chosen as a nonpolar model solvent, since it features structural similarities to the PS repeating unit and a reasonable high boiling point. For these screening experiments, 500 ppm was selected as the concentration to ensure complete solubility at elevated temperatures. The applied self-assembly protocol included dissolving the respective kinked bisamides at elevated temperatures until an optically clear solution was obtained. Subsequent cooling to room temperature resulted in a turbid dispersion indicating supramolecular object formation. Evaporation of the solvent allows for isolation of the supramolecular structures and subsequent characterization by SEM.

**Figure 1:**
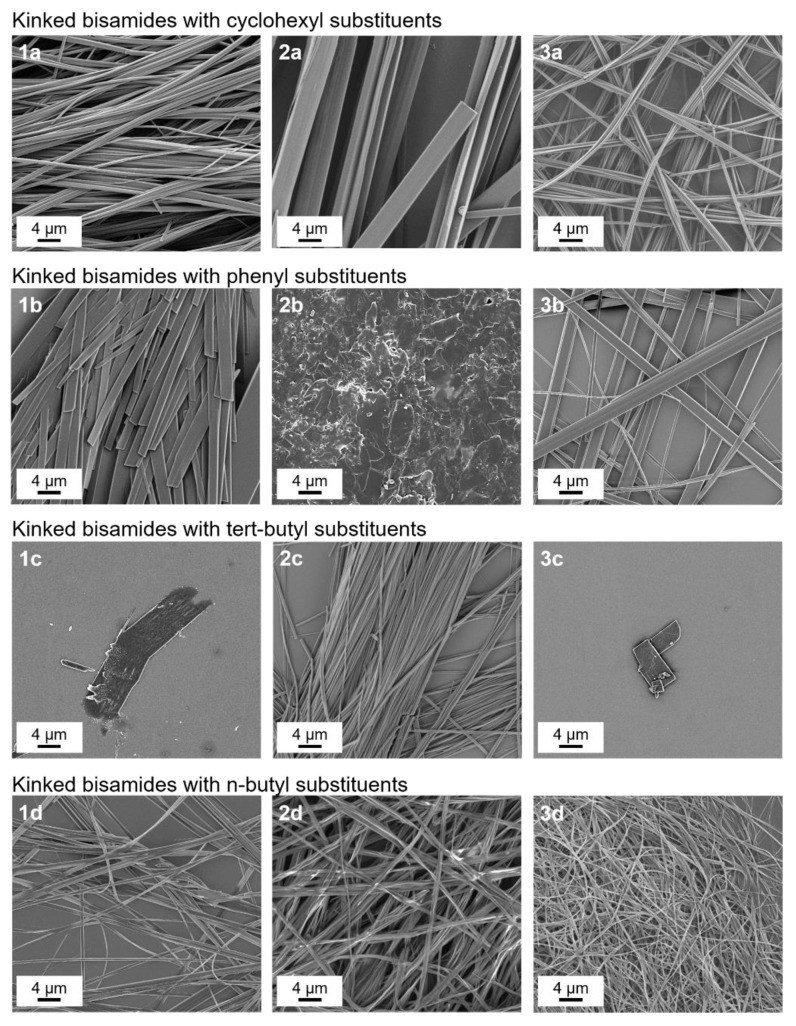
SEM micrographs (2000×) of isolated supramolecular nano-objects of kinked bisamides self-assembled from xylene at an additive concentration of 500 ppm.

An overview of the self-assembled structures of the twelve kinked bisamides is shown in Figure 1. In general, most of the structures are strongly elongated with a fibrillar- or ribbon-like morphology. This indicates that there is at least one preferred direction with a fast growth rate, which is typically driven by the formation of a hydrogen bond pattern. In the case of the kinked bisamides **1a**–**3a** with cyclohexyl substituents in the periphery, a dense network of fibrillar-like structures can be observed. While the fibrillar structures of **1a** exhibit a width in the range of 0.2–1.3 µm, they are in the range of 0.5–4.5 µm for **2a**. The widths of the fibrillar structures of **3a** are between 0.1 and 0.6 µm. The kinked bisamides **1b**–**3b** with phenyl groups as peripheral substituents feature a distinctly different morphology, resembling ribbon- or plate-like structures. The widths of the ribbon-like structures of **1b** and **3b** are in the range of 0.8–3.8 µm and 0.3–2.4 µm, respectively.

In contrast, **2b** forms micrometer-sized less-defined plate-like structures. Kinked bisamides **1c–3c** with tert-butyl groups in the periphery show again supramolecular objects of different morphology. While **1c** and **3c** self-assemble into plate-like structures with large widths between 1.0 μm and 20.0 μm, a dense fibrillary-like network with widths between 0.2 µm and 1.0 µm is obtained for **2c**. All three kinked bisamides **1d–3d** with n-butyl substituents in the periphery build up dense networks of fibrillar structures. The widths of the fibrillar structures are in the ranges of 0.2–1.5 µm for **1d**, 0.3–1.6 µm for **2d**, and 0.1–1.0 µm for **3d**. To conclude, the results of the self-assembly experiments indicate that all kinked bisamides can form small supramolecular nano-objects. In particular, the kinked bisamides **1a–3a** and **1d–3d** show the most promising self-assembled structures in terms of finely dispersed fibrillary objects.

### 3.3. Crystal Structure Solution of ***3a***

**Figure 2:**
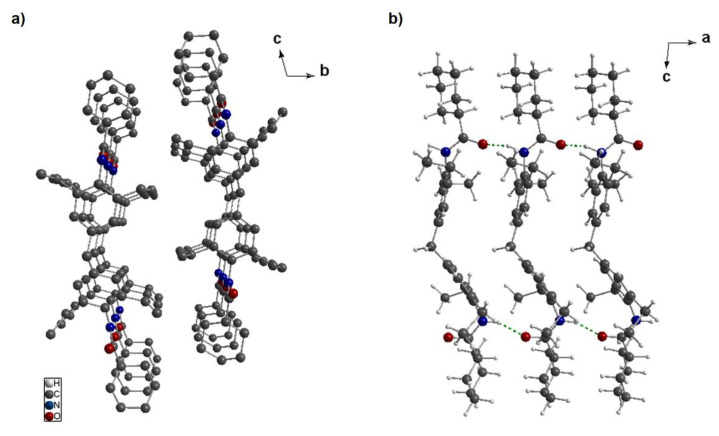
Crystal packaging plots of **3a** recrystallized from acetone. (**a**) A section of the crystal structure of **3a** with a viewing direction along the a-axis (hydrogens were omitted for clarity). The molecules are arranged in a stacking-type manner, whereas each stack comprises two strands of hydrogen bonds along the a-axis. (**b**) A section of the crystal structure of **3a** with a viewing direction along the b-axis. Every molecule forms two ditopic hydrogen bonds (green dotted lines) to two neighboring molecules. Notably, the hydrogen bonds pattern of the two strands point in the opposite direction.

The findings of the self-assembly experiments in a nonpolar solvent demonstrated that most of the kinked bisamides feature very long and fine fibrillar- or ribbon-like structures, which is indicative of a highly preferred directed growth of the structures. In general, the mesoscopic growth and shape of nanostructures is reflected by the molecular structure and the specific pattern of the hydrogen bonds in the crystallographic unit cell. For none of the twelve kinked bisamides, single crystals suitable for single crystal x-ray diffraction could be achieved, which often is the case for compounds yielding supramolecular nanostructures. Thus, we selected a well-ordered crystalline powder of kinked bisamide **3a** recrystallized from the polar solvent acetone suitable to reveal the crystal structure by powder x-ray diffraction (PXRD). The structure was solved using real space methods. Details on the PXRD investigation, simulation, refinement, and solid-state NMR spectroscopy are given in the Supplementary Materials, Section S4 (structure solution) and S5 (solid state NMR). Structure elucidation of the crystalline powder of the kinked bisamide **3a** from acetone features a reasonably low value of R_wp_ of 3.56 after Rietveld refinement, demonstrating a high agreement of the PXRD experiment with the simulation, validating the structure model. It was found that **3a** crystallizes in the triclinic space group $P\bar{1}$, with the parameters a = 4.6882(8) Å, b = 15.4521(19) Å, c = 22.5115(66) Å, α = 105.23(2)°, β = 93.88(4)°, and γ = 93.07(3)°. The triclinic unit cell contains two molecules linked by an inversion center and has a volume of 1565.57(1) Å^3^ as well as a crystallographic density of 1.12591 g/cm^3^. The corresponding crystal packaging plots of kinked bisamide **3a** are depicted in Figure 2. When viewed along the a-axis (Figure 2a), the molecules are arranged in columnar stacks. These columnar stacks comprise two parallel arranged strands of hydrogen bonds along the a-axis, which is most likely the driving force for the elongated or fibrillar-like morphology. A crystal packaging plot of **3a** with a viewing direction along the b-axis is depicted in Figure 2b, showing that the molecules are arranged in an elongated manner along the c-axis. This arrangement resembles a 2D layer with a thickness of a single molecule. Within both directions, along the b-axis as well as the c-axis, the molecules are held together by weaker van der Waals forces, which can be assumed to represent the two other dimensions of the nanostructures yielding under proper conditions a ribbon or plate-like morphology depending on the individual growth rate. Figure 2b also provides a clearer picture of the intermolecular hydrogen bonds pattern of the two strands between the molecules in the crystalline state (green dotted lines). Each amide group forms a ditopic hydrogen bond pattern with the same amide group of the next two neighboring molecules, resulting in total four intermolecular hydrogen bonds per molecule. The average NH··O distances were found to be ≈1.7 Å, which corresponds to a medium strong hydrogen bond [38]. This hydrogen bond length fits the observed proton shift of 8.5 ppm (Figure S4) [39]. Interestingly, the oxygen atoms within a strand of hydrogen bonds in a column point in the opposite direction. Thus, the hydrogen bond pattern formed within a stack is arranged in an antiparallel manner. PXRD on samples of kinked bisamide **3a** self-assembled from xylene results in broad diffraction peaks and could therefore not be used for a structure solution. This clearly indicates that the self-assembled structures of **3a** feature a much higher degree of disorder.

To verify that the fundamental supramolecular motif, that is the arrangement into columnar stacks with two parallel arranged strands of hydrogen bonds, is comparable, we performed solid-state NMR spectroscopy (see the Supplementary Materials, Section S5). It was found that the number of carbon resonances from the ^13^C{^1^H} CP NMR spectrum matches the obtained crystal structure, as there is one molecule in the asymmetric unit resulting in an individual carbon shift for all carbon atoms. Comparing both spectra, the ^1^H and ^13^C{^1^H} CP NMR spectra of **3a** recrystallized from acetone and from xylene show that all chemical shifts and intensities of all resonances are similar; however, the resonances for the sample prepared from xylene are broader. Therefore, we assume the same topology with a similar molecular coordination and self-assembly behavior from xylene. Moreover, we performed FTIR measurements on both samples (see the Supplementary Materials, Section S6). Specific vibrations are very sensitive to strongly bonded hydrogen bonds of amide groups as well as their arrangement in a crystalline packing. This in particular includes amide A (N–H stretch vibrations) and amide II (C–N deformation vibration). The comparison of both spectra shows that they are almost identical particularly in view of the amide-related vibrations. Similarly, as shown before, it was found that the amide A vibration of the xylene sample is slightly broadened. This finding is in agreement with the PXRD and the solid-state NMR spectroscopic results indicating that structural arrangement in the xylene sample is most likely the same, however, with a lower degree of order.

### 3.4. Batch Foaming of Polystyrene Using Kinked Bisamides

**Figure 3:**
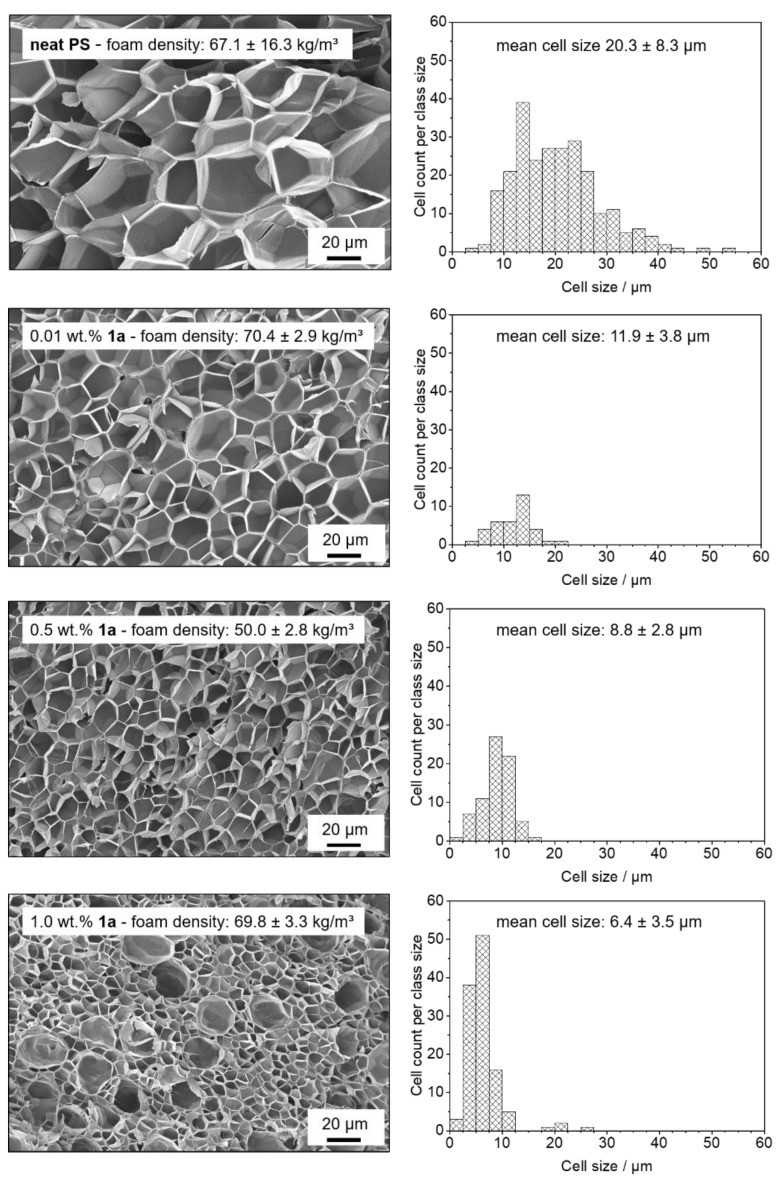
(**Left**): SEM micrographs (500×) depicting the cell morphologies of batch foamed neat PS specimens and batch foamed PS specimens with 0.01, 0.5, and 1.0 wt.% of kinked bisamide **1a**. The mean foam density is given for each concentration. (**Right**): corresponding histograms of the cell sizes including the mean cell sizes.

To evaluate how supramolecular objects of the kinked bisamide can act as foam cell nucleating agents for PS foams, a thermally induced batch foaming process was used as a screening method. The additive concentration was varied in the range from 0.01 to 1.0 wt.% to investigate the concentration-dependent influence of the kinked bisamides on the PS foam properties. All foams were characterized with respect to their cell morphology, the mean cell size, cell density and homogeneity, as well as the foam density. Exemplarily, the SEM micrographs and histograms of the foam cells for PS foams with the kinked bisamide **1a** at concentrations of 0.1, 0.5, and 1.0 wt.% as well as a neat PS reference foam are shown in Figure 3.

Using these conditions, the neat PS reference foam exhibits cell sizes in the range from 5 µm and 55 µm, yielding a mean cell size of 20.3 ± 8.3 µm with a significantly large cell size distribution. The cell density and the foam density of the reference sample was found to be 2.1 × 10^9^ cm^−3^ and 67.1 ± 16.3 kg/m^3^, respectively. PS foams containing only 0.01 wt.% of the kinked bisamide **1a** already exhibits a reduction in the mean cell size to 11.9 ± 3.8 µm including a significant narrower cell size distribution. Whereas the cell density was larger by a factor of 2, the foam density with 70.4 ± 2.9 kg/m^3^ was found to be comparable, demonstrating that the foam density is not influenced significantly by addition of the additive. Upon further increasing the concentration to 0.5 wt.%, the mean cell size is further reduced to 8.8 ± 2.8 µm. The overall morphology of the PS foam is highly homogeneous and does not possess cells with sizes above 20 µm, with the foam density being 50.0 ± 2.8 kg/m^3^. For PS foams with a highest concentration of 1.0 wt.%, a foam density of 69.8 ± 3.3 kg/m^3^ with 20-fold increase in cell density and a mean cell size of 6.4 ± 3.5 µm was achieved, with the latter being noticeably smaller than the values found for the other foams with the kinked bisamide **1a**. However, these foams feature a cell morphology with a distinct inhomogeneity, as is indicated by the slightly increased standard deviation compared to the PS foams with a concentration of 0.5 wt.% of **1a**. As shown in Figure 3, the SEM micrographs as well as the corresponding histogram exhibit a large number of cells with sizes below 10 µm and some cells with sizes in the range of 20 µm. Notably, the large fraction of the smaller sized cells is significantly smaller compared to the PS foams with lower additive concentration. This indicates that a larger number of nucleating sites is still present and lets us assume that there is no severe dissolution and self-assembly issue at a high concentration of **1a**. Although bimodal foam morphologies were reported in the literature [2,40], we cannot clearly assign at the current stage this inhomogeneity to a specific effect caused by the additive.

**Figure 4:**
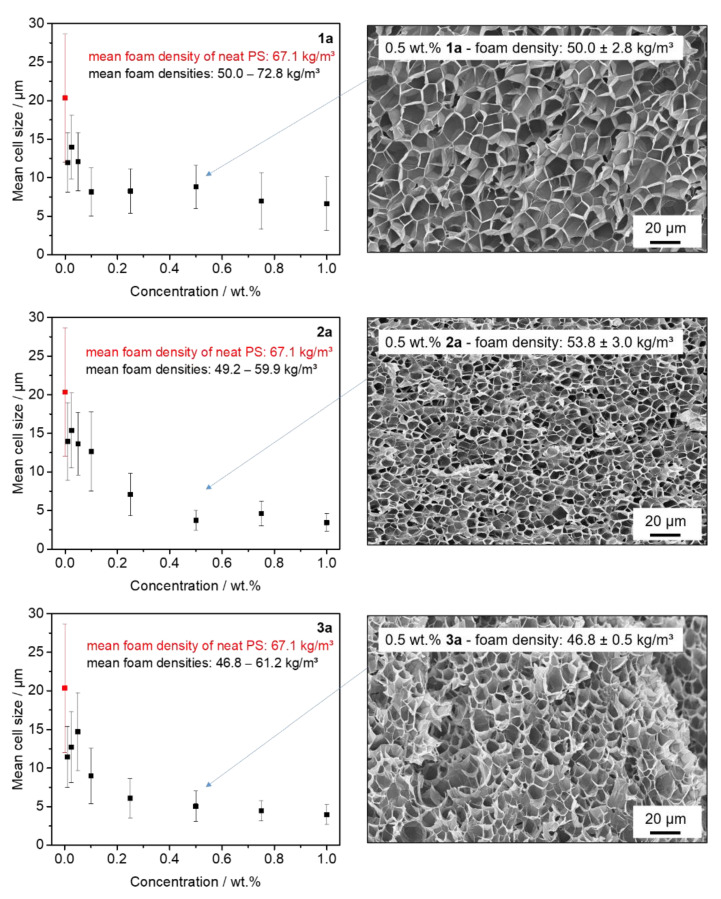
(**Left**): evolution of the mean cell size with increasing concentrations of the kinked bisamides **1a**, **2a**, and **3a** of batch foamed polystyrene specimens. Data for the neat PS reference foam are shown in red. (**Right**): SEM micrographs (500×) depicting the homogeneous microcellular morphology of batch foamed polystyrenes at a concentration of 0.5 wt.% for **1a**, **2a** and **3a**, including the mean foam densities.

To gain further insight into structure–property relations with respect to the molecular design and the cell nucleation capabilities, we compare the concentration-dependent evolution of the foam morphology of **1a** with those foams obtained with **2a** and **3a**. Within this series, the substitution pattern R_1_ is varied from H to methyl to ethyl moieties at the ortho-positions close to the amide units. All of these compounds feature a fibrillary-like morphology when self-assembled from nonpolar solvents yet higher transition temperatures for **2a** and **3a**. After batch foaming of PS with the various concentrations and investigating the foam densities, all foams featured mean foam densities in the range from 46.8 to 72.8 kg/m^3^, corresponding to volume expansion ratios between 13 and 20. Figure 4 shows the concentration-dependent evolution of the mean cell size of PS batch foams with kinked bisamides of **1a**, **2a**, and **3a**. Similarly, as shown above, all kinked bisamides are capable of reducing the mean cell size in the investigated additive concentration range from 0.01 wt.% to 1.0 wt.% significantly. In general, compared to neat PS foams, a reduction in the mean cell size by more than 40% was found at concentrations larger than 0.25 wt.% for all additives. In addition, a significant reduction in the standard deviations of the mean cell sizes was achieved at these concentrations. Interestingly, when comparing the concentration-dependent series of **1a** with **2a** and **3a**, it was found that foams with the latter two additives showed a much more homogenous microcellular morphology and a significant smaller distribution of foam cell sizes. Thus, we conclude that these additives provide more nucleation sites. This is clearly demonstrated in Figure 4 in the SEM micrographs for the concentration of 0.5 wt.% for each kinked bisamide. Moreover, as already indicated by the smaller standard deviation of foams with **2a** and **3a**, no SEM micrograph shows an inhomogeneous cell morphology (see the Supplementary Materials, Section S7). Together with the smaller cells at high concentrations, this finding strongly indicates that there are no dissolution and dispersion issues present using this kind of additive. The PS foams with the finest morphologies were found by using a concentration of 1.0 wt.% of **2a** and **3a** featuring a microcellular morphology with mean cell sizes of 3.5 ± 1.1 µm and 3.9 ± 1.3 µm and cell densities of 5.7 × 10^11^ and 3.5 × 10^11^ cm^−3^, respectively. Thus, compared to the reference foam, the mean cell sizes can be improved by a factor of 5 and the cell densities by two orders of magnitude.

These concentration-dependent batch foam experiments were also performed with the kinked bisamides of **1b**, **2b**, and **3b** comprising peripheral phenyl side groups; the kinked bisamides of **1c**, **2c**, and **3c** with peripheral tert-butyl side groups; and **1d**, **2d**, and **3d** with peripheral n-butyl side groups. For the sake of completeness, all details on the foaming process, concentration-dependent evolution of the mean cell size and representative micrographs are given in the Supplementary Materials, Section S7. A summary of all relevant PS batch foam properties with kinked bisamides are given in the Supplementary Materials, Section S8. In general, all investigated kinked bisamides feature a highly efficient foam cell nucleation capability, resulting in a comparable mean cell size reduction of 30–40%. Moreover, the foam densities of all PS foams remain similar, demonstrated by the volume expansion ratio in the range of 10–20.

Although the different kinked bisamides feature a slightly different morphology of the nanostructures, this has no clear influence on the foam morphology. However, the trend observed for the series **1a**, **2a**, and **3a** can also be found for the other series demonstrating that the substitution pattern R_1_ has a beneficial effect on foam cell nucleation and thus on the microcellular morphology.

### 3.5. Extrusion Foaming of Polystyrene Using Kinked Bisamides

Foam extrusion was selected to demonstrate the applicability of the concept using kinked bisamides as foam cell nucleating agents on a technically relevant continuous process. We selected the kinked bisamide **3a** for these experiments. To perform a concentration-dependent series of experiments with a kinked bisamide and to evaluate suitable parameters on our setup, a total amount of the additive on a 100 g scale is required. Foam extrusion, including material feeding and temperature protocols, was performed in a similar manner as described before (see also the Materials and Methods section) and in References [29,30]. To study the influence of the concentration of **3a** with respect to the foam morphology and density, we prepared PS foams with 0.1, 0.2, and 0.5 wt.% of **3a.** This also provides information on the dissolution and self-assembly behavior of **3a** in the gas-loaded polymer melt under harsher processing conditions such as large shear forces, and high barrel temperature and pressure.

**Figure 5:**
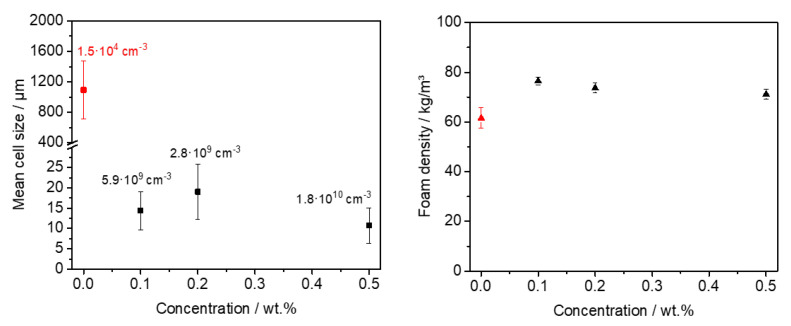
Left: mean cell sizes versus additive concentration of XPS foam specimens nucleated with **3a**. The respective cell densities are added for each concentration. Right: foam densities versus additive concentration of extrusion foamed polystyrene specimens nucleated with **3a**. Data for the XPS reference foam are shown in red.

Similar to the batch foamed samples, we obtained macroscopically comparable extruded polystyrene foams with the kinked bisamide **3a** and the neat PS reference foam (for a photographic image, see the Supplementary Materials, Section S9) featuring low foam densities with volume expansion ratios in the range of 15. In detail, while the foam density of the neat XPS foam was found to be 61.6 ± 4.1 kg/m^3^, the foam densities with **3a** were found to be 76.5 ± 1.5 kg/m^3^ at 0.1 wt.%, 73.7 ± 2.0 kg/m^3^ at 0.2 wt.%, and 71.2 ± 1.9 kg/m^3^ at 0.5 wt.%, respectively (see Figure 5). The neat PS reference foam clearly possesses a coarse macro-cellular foam morphology with large cell sizes and cell size distribution (see Supplementary Materials, Section S10) and determined to be 1094 ± 377 µm. Correspondingly, a cell density of 1.5 × 10^4^ cm^−3^ was calculated. In contrast, XPS foams with the kinked bisamide **3a** applying the same or very similar conditions exhibit significant smaller cell sizes. XPS foams with 0.1 wt.% of **3a** already leads to a 70 times cell size reduction with a mean cell size of 14.4 ± 4.7 µm. Upon further increasing the concentration of **3a** to 0.2 wt.% and 0.5 wt.%, the mean cell sizes were found to be 19.0 ± 6.7 µm and 10.7 ± 4.3 µm, respectively. Thus, using a concentration of 0.5 wt.% of **3a** results in a reduction in the cell size by two orders of magnitude. Expectedly, for the foams with to 0.1 wt.%, 0.2 wt.% and 0.5 wt.% of **3a**, the cell densities were significantly increased by 6 orders of magnitude with respect to the reference foam featuring values of 5.9 × 10^9^ cm^−3^, 2.8 × 10^9^ cm^−3^, and 1.8 × 10^10^ cm^−3^, respectively.

**Figure 6:**
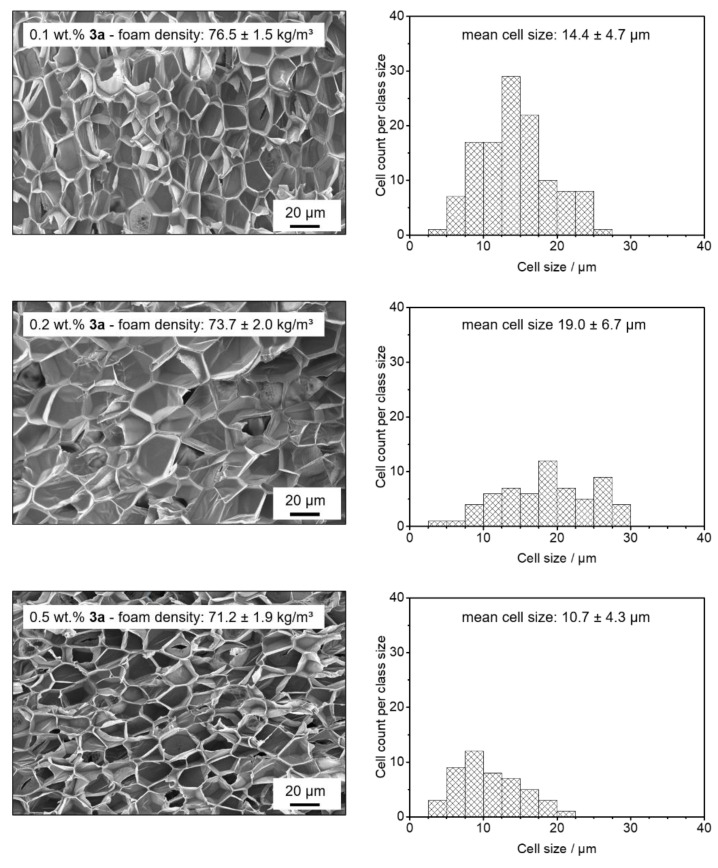
(**Left**): SEM micrographs (500×) of the cell morphologies and mean foam densities of extrusion foamed polystyrene specimens with 0.1 wt.% (top), 0.2 wt.% (middle), and 0.5 wt.% (bottom) of kinked bisamide **3a**, demonstrating the homogeneity of the morphology for all foams. (**Right**): corresponding histograms of the cell sizes including the mean cell sizes.

In Figure 6, SEM micrographs of the cell morphologies of XPS foam specimens with different concentrations of **3a** and the corresponding cell size histograms are shown. The micrographs reveal that no significant portion of an open-cell content can be seen. Moreover, for all concentrations, a homogeneous morphology was found. This is in agreement with the results from the batch-foamed samples demonstrating the suitability of **3a** as a foam cell nucleating agent over a large concentration range. These data strongly suggest that there is no dissolution, self-assembly, or dispersion issue with this class of molecules at the selected conditions. To sum up, the results show that the kinked bisamide **3a** is a very effective foam cell nucleating agent for the foam extrusion even at a concentration of 0.1 wt.%. Interestingly, this class of compounds can be used over a large concentration range without an indication of a dissolution, self-assembly, or dispersion issue. At larger concentrations of 0.5 wt.%, closed small cells in the range of 10 µm can be found without sacrificing the foam density, rendering this material class as highly promising in preparing low-density polystyrene foams with homogeneous microcellular morphology.

## 4. Conclusions

We demonstrated that the class of kinked bisamides, based on various 4,4′-diphenylmethane central units and via amide-linked peripheral side groups, is highly suitable to act as efficient supramolecular foam cell nucleating agents for PS. Systematic variation of the molecular design allows us to tune the thermal properties as well as the mesoscopic morphology of the nano-objects from fibrillary to ribbon to a plate-like shape. Structural elucidation on a selected kinked bisamide reveals that such an elongated fibrillary or ribbon-like shape is attributed to the formation of two strands of hydrogen bonds, which can be regarded as the main driving force for a preferred one-dimensional crystal growth. It was found that all kinked bisamides can be conveniently dissolved at elevated temperatures in a large range of concentrations, which subsequently forms homogeneously dispersed nano-objects upon cooling, providing a large number of nucleation sites for the foam cell nucleating. In a series of batch foaming experiments, we have shown that using 1.0 wt.% of the kinked bisamide with cyclohexane side groups and a methyl substituent in ortho position to the amide groups feature a highly homogeneous cell morphology of the resulting PS foam with a mean cell size as low as 3.5 µm and a foam density of 52 kg/m^3^. Kinked bisamide **3a** with cyclohexane side groups and ethyl substituents at the ortho-positions of the amide groups was selected to study the concentration-depended behavior of the additive in a continuous foam extrusion process. All XPS foams feature comparable foam densities in the range of 67 to 80 kg m^−3^. Using 0.5 wt.% of **3a**, we were able to achieve polymer foams with a homogeneous morphology and cell sizes in the range of 10 µm, which is lower by a factor 100 and a cell density of ≈10^10^ cm^−3^, which is higher by 6 orders of magnitude compared to the neat PS reference foam. These findings demonstrate that the class of kinked bisamides is a promising candidate to achieve microcellular PS foams with a defined microcellular morphology, a regime in which the Knudsen effect becomes relevant.

## Figures and Tables

**Table 1:**
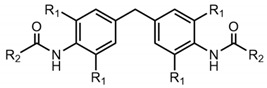
Structural variation of the kinked bisamides **1a**–**3d** at the ortho-positions to the amide groups R_1_ and the peripheral side groups R_2_.

	R2	Cyclohexyl	Phenyl	Tert-Butyl	N-Butyl
R1					
H		1a	1b	1c	1d
methyl		2a	2b	2b	2d
ethyl		3a	3b	3b	3d

## Data Availability

The presented data are available by the authors upon request.

## Supplementary Materials

The following are available online at https://www.mdpi.com/article/10.3390/polym13071094/s1, Section S1: Schematic representation of foam cell nucleation with supramolecular nanoobjects, Section S2: Synthesis and characterization of kinked bisamides, Section S3: Thermal properties of kinked bisamides, Section S4: PXRD investigation of kinked bisamide **3a**, Section S5: Solid-state NMR spectroscopy of kinked bisamide **3a**, Section S6: FT-IR spectroscopy of kinked bisamide **3a**, Section S7: Cell morphologies of polystyrene foams by batch foaming with kinked bisamides, Section S8: Summary of polystyrene batch foam properties with kinked bisamides, Section S9: Photograph of an extruded neat polystyrene foam and a polystyrene foam with 0.5 wt.% **3a**, Section S10: Photograph and light microscopy image of neat polystyrene foams by extrusion foaming. A crystallographic information file (CIF) is provided as additional supporting information.
