# Supplementary material for: Kinked Bisamides as Efficient Supramolecular Foam Cell Nucleating Agents for Low-Density Polystyrene Foams with Homogeneous Microcellular Morphology
Source: Polymers (Basel). 2021 Mar 30;13(7):1094. doi: 10.3390/polym13071094 (PMC8036985; doi:10.3390/polym13071094)
Supplement: Supplementary file 1 [file polymers-13-01094-s001.pdf]

polymers

Supporting Information

Kinked Bisamides as Efficient Supramolecular Foam Cell Nucleating Agents for Low-Density Polystyrene Foams With Homogeneous Microcellular Morphology

Bastian Klose [1], Daniel Kremer [1], Merve Aksit [2], Kasper P. van der Zwan [3], Klaus Kreger [1], Jürgen Senker [3,4,*], Volker Altstädt [2,4,*] and Hans-Werner Schmidt [1,4,*]

1 Macromolecular Chemistry I, University of Bayreuth, Universitaetsstrasse 30, 95447 Bayreuth, Germany; bastian.klose@uni-bayreuth.de (B.K.); daniel.kremer@uni-bayreuth.de (D.K.); klaus.kreger@uni-bayreuth.de (K.K.); hans-werner.schmidt@uni-bayreuth.de (H.-W.S.)
2 Department of Polymer Engineering, University of Bayreuth, Universitaetsstrasse 30, 95447 Bayreuth, Germany; merve.aksit@uni-bayreuth.de (M.A.); altstaedt@uni-bayreuth.de (V.A.)
3 Inorganic Chemistry III, University of Bayreuth, Universitaetsstrasse 30, 95447 Bayreuth, Germany; kasper.van-der-Zwan@uni-bayreuth.de (K.P.Z.); juergen.senker@uni-bayreuth.de (J.S.)
4 Bavarian Polymer Institute and Bayreuth Institute of Macromolecular Research, University of Bayreuth, Universitaetsstrasse 30, 95447 Bayreuth, Germany
* Correspondence: hans-werner.schmidt@uni-bayreuth.de; altstaedt@uni-bayreuth.de; juergen.senker@uni-bayreuth.de; Tel.: +49 921553200 (H.-W.S.); +49 921557471 (V.A.); +49 921552532 (J.S.)

Table of content

S1. Schematic representation of foam cell nucleation with supramolecular nanoobjects

S2. Synthesis and characterization of kinked bisamides

S3. Thermal properties of kinked bisamides

S4. PXRD investigation of kinked bisamide 3a

S5. Solid-state NMR spectroscopy of kinked bisamide 3a

S6. FTIR spectroscopy of kinked bisamide 3a

S7. Cell morphologies of polystyrene foams by batch foaming with kinked bisamides

S8. Summary of polystyrene batch foam properties with kinked bisamides

S9. Photograph of an extruded neat polystyrene foam and a polystyrene foam with 0.5 wt.% 3a

S10. Photograph and light microscopy image of neat polystyrene foams by extrusion foaming

References

S1. Schematic representation of foam cell nucleation with supramolecular nanoobjects

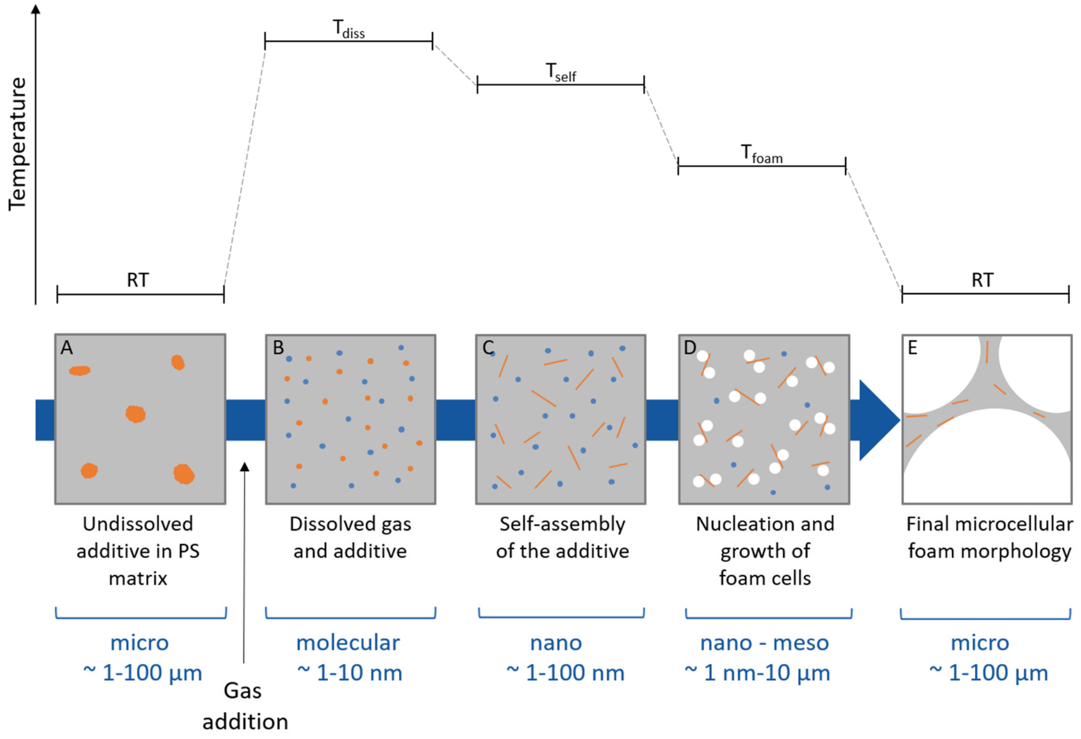

Figure S1. Schematic representation of the generalized process using supramolecular nanoobjects as heterogeneous cell nucleation agents for polymer foaming. The temperature and the relevant length scale, at which each individual step occurs, is indicated. The conceptual approach comprises: A) The preparation of a powder-powder mixture with the finely micro-sized supramolecular additive. B) Upon heating, the supramolecular additive is molecularly dissolved and both the additive as well as the added physical blowing agent is homogeneously dispersed in the polymer melt. C) Upon cooling, self-assembly of the supramolecular additive results into finely dispersed in-situ formed nanoobjects. D) Upon further cooling, these nanoobjects acts as heterogeneous nucleation sites for the foam cells, which subsequently grows during the foaming step on the nano and mesoscale. E) At room temperature, a solid foam with a microcellular foam morphology is obtained.

S2. Synthesis and characterization of kinked bisamides

General synthetic procedure

1 eq. of 4,4'-Diaminodiphenylmethane, 4,4'-Methylenebis(2,6-dimethylaniline) or 4,4'-Methylenebis(2,6-diethylaniline) was added under nitrogen to a mixture consisting of N-methylpyrrolidone (NMP) or tetrahydrofurane (THF) and pyridine or triethylamine (Figure S2). A small amount of LiCl was added and the solution was cooled to 0 – 5 °C. 2.2 eq. of the respective acid chloride was added dropwise and the reaction mixture was heated to 20 – 80 °C. The reaction mixture maintained at this temperature for several hours. After cooling, the reaction mixture was precipitated in ice water under vigorous stirring. The precipitate was filtered off and dried under vacuum for 12h. All compounds were further purified by recrystallization.

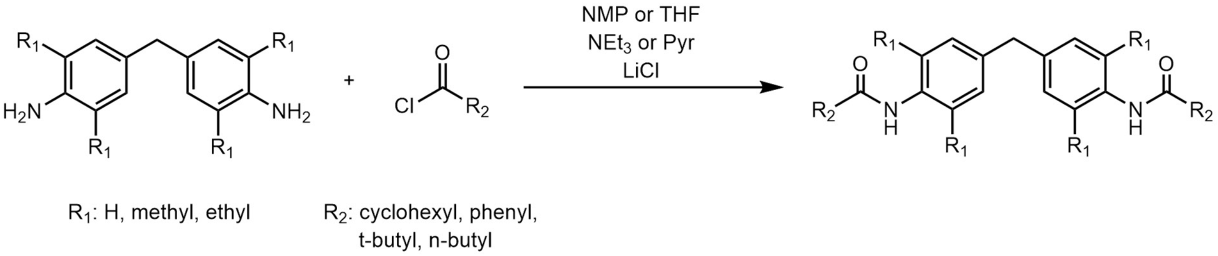

Figure S2. Schematic of the synthetic procedure of N,N′-[Methylenebis(4,1-phenylene)]bisamides.

Molecular characterization

^{1}H-NMR spectra in solution were recorded on a Bruker Avance Ultrashield 300 (300 MHz) at room temperature. For the preparation of the NMR samples, approx. 5 mg of the compounds were dissolved in 1.2 mL of the respective deuterated solvent.

Mass spectra (MS) were carried out on a FINNIGAN MAT 8500 spectrometer from Thermo-Fisher Scientific using electron spray ionization.

HPLC analyses were performed using an Agilent 1100 equipped with a C18 column Agilent Eclipse Plus (d = 3.5 µm, 4.6 x 150 mm). 0.1 mg – 1.0 mg of the compounds were dissolved in 10 mL acetonitrile or NMP at 60 °C. 25 µL of the solutions were injected. As mobile phase an acetonitrile:water mixture (90:10 v/v %) was used with a flowrate of 0.3 mL/min. Detections were performed using a UV-detector at 250 nm and the peak values of the elution volume were reported.

Synthesis of N,N′-[Methylenebis(4,1-phenylene)]bis[cyclohexanecarboxamide] ***1a:*** 3.0 g (15.0 mmol) 4,4′-diaminodiphenylmethane, 4.9 g (33.0 mmol) cyclohexanecarbonyl chloride, 100 mL NMP, 20.0 mL pyridine, 0.1 g LiCl. Reaction conditions: 12 h, 80 °C. Recrystallization from 500 mL MeOH yielded 5.5 g (13.1 mmol, 88 %) of a white solid powder.
^{1}H-NMR (DMSO-d_6, 300 MHz, δ in ppm): 9.73 (2H, s, NH), 7.49 (4H, d, H_{Ar}), 7.08 (4H, d, H_{Ar}), 3.79 (2H, s, Ar-C*H*2-Ar), 2.32-2.25 (2H, m, CH_{Cyc}), 1.75-1.20 (20H, m, $CH2_{Cyc}$). MS-EI (m/z, %): 418 (M+, 100), 309 (46), 308 (100), 199 (14), 198 (95), 197 (20), 106 (17), 83 (42), 55 (32). HPLC (El.-Vol., area in %): 2.68 mL (96).

Synthesis of N,N′-[Methylenebis(4,1-phenylene)]bis[benzamide] ***1b***: 5.0 g (25.2 mmol) 4,4′-diaminodiphenylmethane, 7.8 g (55.4 mmol) benzoyl chloride, 100 mL NMP, 4.5 mL pyridine. Reaction conditions: 1 h, 20 °C. Boiling in 500 mL MeOH yielded 9.6 g (23.7 mmol, 95%) of a white solid powder.
^{1}H-NMR (DMSO-d_6, 300 MHz, δ in ppm): 10.21 (2H, s, NH), 7.95-7.92 (4H, m, H_{Ar}), 7.70 (4H, d, H_{Ar}), 7.60-7.49 (6H, m, H_{Ar}), 7.20 (4H, d, H_{Ar}), 3.89 (2H, s, Ar-C*H*2-Ar). MS-EI (m/z, %): 406 (M+, 100), 301 (36), 210 (11), 106 (58), 105 (98), 78 (23), 77 (98), 51 (55). HPLC (El.-Vol., area in %): 2.07 mL (100).

Synthesis of N,N′-[Methylenebis(4,1-phenylene)]bis[tertbutylcarboxamide] ***1c***: 3.0 g (15.0 mmol) 4,4′-diaminodiphenylmethane, 4.0 g (33.0 mmol) pivaloyl chloride, 100 mL NMP, 20.0 mL pyridine, 0.1 g LiCl. Reaction conditions: 24 h, 80 °C. Recrystallization from 500 mL ethyl acetate yielded 4.1 g (11.1 mmol, 75 %) of a white solid powder.
^{1}H-NMR ($CDCl_3$, 300 MHz, δ in ppm): 7.45 (2H, d, H_{Ar}), 7.28 (2H, m, NH), 7.13 (2H, d, H_{Ar}), 3.92 (2H, s, Ar-C*H*2-Ar), 1.32 (18H, s, CH3). MS-EI (m/z, %): 366 (M+, 100), 309 (15), 283 (15), 282 (64), 198 (11), 197 (16), 132 (10), 58 (91), 42 (12). HPLC (El.-Vol., area in %): 2.17 mL (100).

Synthesis of N,N′-[Methylenebis(4,1-phenylene)]bis[pentanamide] ***1d:*** 5.0 g (25.2 mmol) 4,4′-diaminodiphenylmethane, 6.0 g (55.5 mmol) valeroyl chloride, 100 mL NMP, 4.5 mL pyridine. Reaction conditions: 1 h, 20 °C. Recrystallization from 300 mL MeOH yielded 8.8 g (24.0 mmol, 95 %) of a white solid powder.

^{1}H-NMR (DMSO-d$_6$, 300 MHz, δ in ppm): 9.80 (2H, s, NH), 7.48 (4H, d, H$_{Ar}$), 7.10 (4H, d, H$_{Ar}$), 3.80 (2H, s, Ar-C*H*2-Ar), 2.29-2.24 (4H, t, CO-CH2), 1.58-1.53 (4H, quint, CH2-C*H2*), 1.34-1.29 (4H, sext, *CH2*-CH3), 0.91-0.86 (6H, t, CH2-C*H3*). MS-EI (m/z, %): 366 (M+, 99), 324 (22), 323 (20), 283 (48), 282 (100), 240 (18), 199 (24), 198 (100), 197 (53), 182 (19), 180 (12), 132 (15), 106 (44), 57 (31), 41 (16). HPLC (El.-Vol., area in %): 2.14 mL (100).

Synthesis of N,N′-[Methylenebis(2,6-dimethyl-4,1-phenylene)]bis[cyclohexanecarboxamide] ***2a:*** 3.6 g (14.0 mmol) 4,4′-methylenebis(2,6-dimethylaniline), 4.5 g (30.8 mmol) cyclohexanecarbonyl chloride, 100 mL THF (dried), 4.3 mL triethylamine. Reaction conditions: 48 h, 60 °C. Recrystallization from 250 mL DMF yielded 5.6 g (11.8 mmol, 84 %) of a white solid powder.

^{1}H-NMR (DMSO-d$_6$, 300 MHz, δ in ppm): 8.99 (2H, s, NH), 6.88 (4H, s, H$_{Ar}$), 3.73 (2H, s, Ar-C*H*2-Ar), 2.38-2.26 (2H, m, CHCyc), 2.05 (12H, s, CH3), 1.84-1.15 (20H, m, CH2Cyc). MS-EI (m/z, %): 474 (M+, 99), 391 (12), 366 (10), 365 (67), 364 (100), 255 (11), 254 (45), 253 (10), 239 (15), 132 (18), 83 (40), 55 (29). HPLC (El.-Vol., area in %): 2.75 mL (94).

Synthesis of N,N′-[Methylenebis(2,6-dimethyl-4,1-phenylene)]bis[benzamide] ***2b:*** 5.0 g (19.7 mmol) 4,4′-methylenebis(2,6-dimethylaniline), 6.1 g (43.2 mmol) benzoyl chloride, 100 mL NMP, 3.5 mL pyridine. Reaction conditions: 1 h, 20 °C. Boiling in 500 mL MeOH yielded 7.5 g (16.2 mmol, 82 %) of a white solid powder/crystals.

^{1}H-NMR (DMSO-d$_6$, 300 MHz, δ in ppm): 9.70 (2H, s, NH), 7.99-7.97 (4H, m, H$_{Ar}$), 7.55-7.52 (6H, m, H$_{Ar}$), 6.99 (4H, s, H$_{Ar}$), 3.84 (2H, s, Ar-C*H*2-Ar), 2.15 (12H, s, CH3). MS-EI (m/z, %): 463 (M+, 61), 462 (98), 434 (20), 359 (17), 358 (27), 357 (90), 106 (27), 105 (100), 77 (80). HPLC (El.-Vol., area in %): 2.08 mL (99).

Synthesis of N,N′-[Methylenebis(2,6-dimethyl-4,1-phenylene)]bis[tertbutylcarboxamide] ***2c:*** 4.0 g (15.7 mmol) 4,4′-methylenebis(2,6-dimethylaniline), 4.2 g (34.6 mmol) pivaloyl chloride, 100 mL THF (dried), 2.8 mL pyridine. Reaction conditions: 1 h, 20 °C. The crude product was recrystallized from 100 mL MeOH, filtration of the solid product and drying. Filtration of the dissolved product in DMF over silica gel, subsequent precipitation in water and drying yielded 4.9 g (11.6 mmol, 74 %) of a white solid powder.

^{1}H-NMR (DMSO-d$_6$, 300 MHz, δ in ppm): 8.74 (2H, s, NH), 6.89 (4H, s, H$_{Ar}$), 3.77 (2H, s, Ar-C*H*2-Ar), 2.05 (12H, s, Ar-C*H*3), 1.22 (18H, s, CH3). MS-EI (m/z, %): 423 (M+, 63), 422 (98), 366 (22), 365 (80), 339 (87), 338 (100), 337 (29), 323 (22), 254 (11), 253 (13), 239 (17), 238 (15), 237 (12), 222 (11), 160 (47), 134 (15), 57 (100), 41 (19.5). HPLC (El.-Vol., area in %): 2.24 mL (96).

Synthesis of N,N′-[Methylenebis(2,6-dimethyl-4,1-phenylene)]bis[pentanamide] ***2d:*** 3.0 g (11.8 mmol) 4,4′-methylenebis(2,6-dimethylaniline), 2.8 g (25.9 mmol) valeroyl chloride, 200 mL THF (dried), 2.4 mL pyridine. Reaction conditions: 1 h, 20 °C. Recrystallization from 300 mL MeOH yielded 3.6 g (8.5 mmol, 72 %) of a white solid powder.

^{1}H-NMR (DMSO-d$_6$, 300 MHz, δ in ppm): 9.09 (2H, s, NH), 6.89 (4H, s, H$_{Ar}$), 3.74 (2H, s, Ar-C*H*2-Ar), 2.31-2.26 (4H, t, CO-CH2), 2.07 (12H, s, Ar-C*H*3), 1.63-1.54 (4H, quint, CH2-C*H2*), 1.41-1.28 (4H, sext, *CH2*-CH3), 0.93-0.89 (6H, t, CH2-C*H3*). MS-EI (m/z, %): 423 (M+, 54), 422 (100), 380 (23), 365 (12), 339 (98), 338 (100), 337 (29), 323 (15), 296 (31), 255 (26), 254 (94), 253 (37), 240 (14), 239 (59), 237 (13), 223 (12), 222 (14), 160 (20), 134 (52), 57 (14), 41 (16). HPLC (El.-Vol., area in %): 2.17 mL (99).

Synthesis of N,N′-[Methylenebis(2,6-diethyl-4,1-phenylene)]bis[cyclohexanecarboxamide] ***3a:*** 9.0 g (28.0 mmol) 4,4′-methylenebis(2,6-diethylaniline), 9.4 g (63.0 mmol) cyclohexanecarbonyl chloride, 120 mL NMP, 5.1 mL pyridine. Reaction conditions: 2 h, 20 °C. Boiling in 200 mL acetone yielded 14.2 g (26.7 mmol, 92 %) of a white solid powder.

^{1}H-NMR ($CDCl_3/CF_3COOD$, 300 MHz, δ in ppm): 6.95 (4H, m, H_{Ar}), 3.90 (2H, m, Ar-C*H*2-Ar), 2.61-2.53 (2H, m, CH_{Cyc}), 2.51-2.43 (8H, m, C*H*2-CH3), 2.15–1.26 (20H, m, $CH2_{Cyc}$), 1.15-1.10 (12H, m, CH2-C*H*3). MS-EI (m/z, %): 531 (M+, 42), 530 (97), 448 (40), 447 (100), 421 (24), 420 (85), 419 (41), 310 (11), 309 (10), 293 (12), 281 (13), 188 (26), 162 (37), 160 (43), 83 (41), 55 (29). HPLC (El.-Vol., area in %): 2.49 mL (95).

Synthesis of N,N′-[Methylenebis(2,6-diethyl-4,1-phenylene)]bis[benzamide] ***3b:*** 5.0 g (16.1 mmol) 4,4′-methylenebis(2,6-diethylaniline), 5.0 g (35.5 mmol) benzoyl chloride, 100 mL NMP, 2.8 mL pyridine. Reaction conditions: 1 h, 20 °C. Boiling in 500 mL MeOH yielded 7.0 g (13.5 mmol, 83 %) of a white solid powder.

^{1}H-NMR (DMSO-d_6, 300 MHz, δ in ppm): 9.69 (2H, s, NH), 7.99-7.96 (4H, m, H_{Ar}), 7.59-7.50 (6H, m, H_{Ar}), 7.04 (4H, s, H_{Ar}), 3.94 (2H, s, Ar-C*H*2-Ar), 2.52-2.49 (8H, s, C*H*2-CH3), 1.09 (12H, t, CH3). MS-EI (m/z, %): 519 (M+, 34), 518 (86), 414 (24), 413 (70), 161 (11), 160 (77), 106 (15), 105 (100), 77 (34). HPLC (El.-Vol., area in %): 3.75 mL (99).

Synthesis of N,N′-[Methylenebis(2,6-diethyl-4,1-phenylene)]bis[tertbutylcarboxamide] - ***3c:*** 4.3 g (14.0 mmol) 4,4′-methylenebis(2,6-diethylaniline), 3.7 g (30.7 mmol) pivaloyl chloride, 50 mL THF (dried), 4.3 mL triethylamine. Reaction conditions: 48 h, 60 °C. Recrystallization from 500 mL MeOH and subsequent recrystallization from 250 ml DMF yielded 4.8 g (10.1 mmol, 71 %) of a white solid powder.

^{1}H-NMR (DMSO-d_6, 300 MHz, δ in ppm): 8.74 (2H, s, NH), 6,93 (4H, s, H_{Ar}), 3.86 (2H, s, Ar-C*H*2-Ar), 2.41 (8H, q, C*H*2-CH3), 1.22 (18H, s, CH3), 1.04 (12H, t, CH2-C*H*3). MS-EI (m/z, %): 479 (M+, 24.8), 478 (71), 449 (13), 422 (54), 421 (100), 394 (29), 393 (24), 188 (64), 162 (16), 160 (36), 57 (66). HPLC (El.-Vol., area in %.): 2.89 mL (99).

Synthesis of N,N′-[Methylenebis(2,6-diethyl-4,1-phenylene)]bis[pentanamide] - ***3d:*** 4.3 g (14.0 mmol) 4,4′-methylenebis(2,6-diethylaniline), 3.7 g (30.8 mmol) valeroyl chloride, 50 mL THF (dried), 4.3 mL triethylamine. Reaction conditions: 3 h, 50 °C. Recrystallization from 200 mL MeOH yielded 3.7 g (7.7 mmol, 56 %) of a white solid powder.

^{1}H-NMR (DMSO-d_6, 300 MHz, δ in ppm): 9.08 (2H, s, NH), 6.93 (4H, s, H_{Ar}), 3.83 (2H, s, Ar-C*H*2-Ar), 2.42 (8H, q, C*H*2-CH3), 2.29 (4H, t, CO-CH2), 1.58 (4H, quint, CH2-C*H*2-CH2), 1.35 (4H, sext, *CH2*-CH3), 1.04 (12H, t, CH2-C*H3*), 0.91 (6H, t, CH2-CH2-C*H3*). MS-EI (m/z, %): 479 (M+, 43), 478 (100), 460 (10), 422 (15), 421 (49), 395 (26), 394 (96), 393 (64), 310 (11), 309 (18), 293 (15), 281 (20), 188 (22), 162 (49), 161 (10), 160 (58), 57 (26), 41 (11). HPLC (El.-Vol., area in %): 2.67 mL (88).

S3. Thermal properties of kinked bisamides

To reveal their thermal behavior at elevated temperatures and the phase transitions, the kinked bisamides were investigated by means of thermogravimetric analysis (TGA) and differential scanning calorimeter (DSC) under nitrogen atmosphere.

Thermal characterization methods

For TGA, an automated Mettler Toledo TGA/DSC 3+ operated under nitrogen atmosphere was used to determine the thermal behavior and the weight loss of the kinked bisamides. About 5 to 10 mg of the kinked bisamides were weighed in Al_2O_3 pans and heated from 50 °C to 700 °C at a rate of 10 K/min under nitrogen (60 mL/min). $T_{-5wt.\%}$ value corresponds to the temperature at which 5 wt.% of the sample is lost.

Thermal phase behavior (melting points and crystallization behavior of the kinked bisamides) were determined by means of DSC. Here, an automated Mettler Toledo DSC 2/3 operated under nitrogen atmosphere was used. About 6 to 12 mg of the compounds were weighed into 30 µL high pressure crucible pans. The phase transitions for compounds **1a – 1d**, **2b**, **2d**, **3b** and **3d** were determined in a temperature range of 25 to 300 °C and for the compounds **2a**, **2c**, **3a** and **3c** in a temperature range of 25 to 350 °C, respectively. All measurements were performed with a heating and cooling rate of 10 K/min. Every heating and cooling scan was repeated three times.

Discussion of the thermal behavior

TGA experiments determine the temperature at which mass loss occurs. Mass loss can be either attributed to decomposition of the compounds or sublimation and evaporation, which should be avoided during polymer processing to ensure functioning of the self-assembly process and to keep the concentration constant. TGA reveals that all kinked bisamides show no significant mass loss at temperatures below 290 °C with $T_{-5wt.\%}$ values being between 293 °C (**3d**) and 408 °C (**1b**). This finding renders all kinked bisamides in general suitable to be used as additives during processing of the polystyrene melt. Equally important are the temperatures at which phase transitions of the compounds in bulk occur, because the melting points as well as the crystallization temperatures can be regarded as a first approximation at which temperature the kinked bisamides dissolve in the polystyrene melt and at which temperature the supramolecular building blocks may self-assemble into nano-objects. In particular, the latter has to take place at a temperature before the foaming of the polymer melt is performed demanding reasonable high crystallization temperatures. It was found that all kinked bisamides show high melting transitions in the range of 192 °C (**1d**) to 328 °C (**3c**). Considering the kinked bisamides with aliphatic peripheral side groups, cyclohexyl **(a)**, tert-butyl **(c)** and n-butyl **(d)**, a clear trend can be observed with respect to the melting and crystallization behavior: Derivatives of the series with unsubstituted aromatic central units (**1**) show lower melting transitions than their derivatives with methyl- (**2**) and ethyl (**3**) substituents at the ortho-position of the amide groups. This is attributed to a torsion of amide groups, which leads to a stronger H bonding between the different amides causing a higher melting temperature. Within this series, the bisamides of category **2** and **3** with cyclohexyl (**a**) and t-butyl (**c**) substituents exhibit the highest melting transitions with temperatures of 307 °C (**2a**), 311 °C (**2c**), 297 °C (**3a**) and 328 °C (**3c**). This difference is attributed to the comparable stiff and rigid peripheral side groups in contrast to the series with the rather flexible n-butyl side groups. For kinked bisamide **3c** it should be noted that the melting temperature is very close to the $T_{-5wt.\%}$ value

of 320 °C indicating also an evaporation or decomposition at this temperature. All kinked bisamides with phenyl substituents (**b**) exhibit reasonable high melting temperatures in the range of 225 °C to 258 °C without featuring a clear trend, which may be attributed to the partial conjugation over the amide group rendering a comparison complicated. The $T_{-5wt.\%}$ values from TGA and the peak values of the endothermic transitions from the second heating and cooling run (DSC) are summarized in Table S1.

Summary of thermal properties

Table S1. Thermal properties of the kinked bisamides including melting points and crystallization temperatures (peak values) and $T_{-5wt.\%}$ values.

**Compound**	**R_1**	**R_2**	**Melting Point [°C][a, b)]**	**Crystallization [°C][d)]**	**$T_{-5wt.\%}$ [°C][a)]**
**1a**	H	cyclohexyl	222	173	380
**2a**	methyl	cyclohexyl	307	285	369
**3a**	ethyl	cyclohexyl	297	266	347
**1b**	H	phenyl	249	216	408
**2b**	methyl	phenyl	225	206 [e]	401
**3b**	ethyl	phenyl	258	234	389
**1c**	H	tert-butyl	239	176	320
**2c**	methyl	tert-butyl	311	251	323
**3c**	ethyl	tert-butyl	328	307	320
**1d**	H	n-butyl	192	183	351
**2d**	methyl	n-butyl	190, 257 [c]	252	339
**3d**	ethyl	n-butyl	121, 220 [c]	86, 199, 210 [f]	293

[a] Upon heating, heating rate 10 K/min, under N_2
[b] Second heating scan
[c] Polymorphic melting behavior
[d] First cooling run, cooling rate 10 K/min, under N_2
[e] Value refers to recrystallization upon heating of the second heating scan
[f] Polymorphic crystallization behavior

S4. PXRD investigation of kinked bisamide 3a

Recrystallization of kinked bisamide **3a** from acetone (see section S1) yielded a crystalline white powder with sufficiently high order and was therefore subjected to powder X-ray diffraction (PXRD). A sample of **3a** was filled in a capillary tube with a diameter of 0.5 mm and a tube wall thickness of 0.1 mm. PXRD measurements were carried out in Debye–Scherrer geometry on a STOE StadiP diffractometer using a Cu Kα_1 radiation (λ= 1.5406 Å) and a curved germanium monochromator (oriented according to the 111 plane). The measurements were performed in the 2Θ range of 3 – 50° with a step size of 0.015°.

Prior to the refinement of the crystal structure, the molecular structure of **3a** was optimised using DFT methods with the CASTEP code [1]. Powder indexing and Pawley refinement was done with the programme TOPAS [2]. Simulated annealing with a rotational freedom of the ethyl substituents in ortho-position to the amide units and the cyclohexyl side-groups was performed with the Endeavour programme [3]. Moreover, additional rotational freemdoms were allowed for the C_{Ar}-CH_2-C_{Ar} bonds. The obtained solution was again geometry-refined with DFT methods. A final Rietveld refinement was done with TOPAS. An R_{wp} of 3.56 has been obtained, a preferred orientation of the 4th order using spherical harmonics has been applied [4]. The x-ray diffractogram together with the Rietveld profile plot and the difference of the two for **3a** recrystallized from acetone is shown in Figure S3. The structural elucidation reveals that the kinked bisamide **3a** crystallizes in the triclinic space group $P\bar{1}$ with the parameters a = 4.6882(8) Å, b = 15.4521(19) Å, c = 22.5115(66) Å, α = 105.23(2)°, β = 93.88(4)°, γ = 93.07(3)°. All relevant crystallographic data are summarized in Table S2. A CIF (crystallographic information file) is provided as additional supporting information.

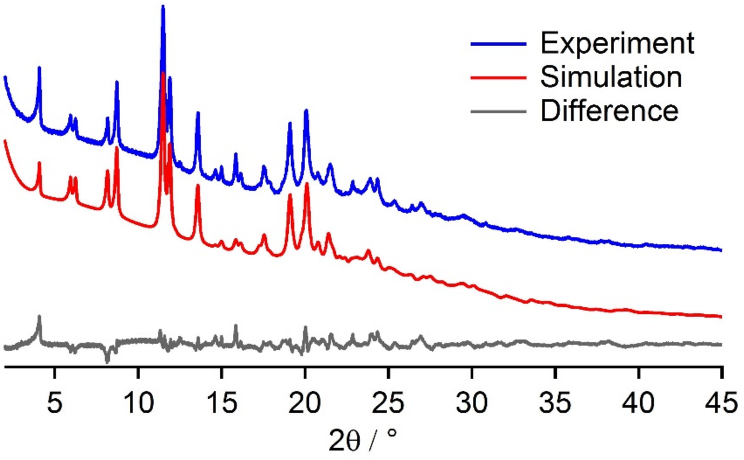

Figure S3. X-ray diffraction pattern (blue) together with the Rietveld profile plot (red) and the difference plot (grey) of **3a** recrystallized from acetone.

Table S2. Crystallographic data of kinked bisamide **3a**.

Crystal System/ Space group	a/Å	b/Å	c/Å	α/°	β/°	γ/°	V/Å^3	Z′/ Z	ρ/g cm^{-3}	T/ K	R_{wp}
Triclinic /$P\bar{1}$	4.6882 (8)	15.4521 (19)	22.5115 (66)	105.23 (2)	93.88 (4)	93.07 (3)	1565.57 (1)	1/2	1.125 91	293	3.56 (3)

S5. Solid-state NMR spectroscopy of kinked bisamide 3a

Solid-state NMR spectroscopy measurements have been performed on recrystallized samples from acetone (see section S1) and self-assembled samples from xylene of kinked bisamide **3a**. To prepare the latter, 500 ppm of **3a** was molecularly dissolved in xylene by refluxing the mixture for 30 minutes. Subsequent cooling to room temperature yielded a turbid dispersion. Evaporation of the solvent under high vacuum yielded a white powder, which was used for the measurement.

Solid-state NMR spectroscopy has been performed on a Bruker Avance III spectrometer operating at a proton resonance frequency of 400 MHz. The two differently prepared samples were packed in 3.2 mm zirconia rotors and analysed in 3.2 mm triple resonance probe. High resolution proton NMR spectra have been recorded under Magic-Angle-Spinning (MAS) with a spinning frequency of 11.5 kHz using the DUMBO decoupling sequence [5]. The ^{13}C NMR spectra were acquired with a cross polarisation pulse programme at 12.5 kHz MAS frequency. The ^{13}C{^{1}H} CP NMR experiments were done with a ramped CP sequence where the nutation frequency on the ^{1}H channel was varied linearly from 50 – 100% [6]. The maximum nutation frequency during the contact time was set to 59 kHz. During acquisition on the ^{13}C channel, ^{1}H were decoupled using the Spinal64 decoupling sequence with a nutation frequency of 68 kHz [7]. The CP contact time was set to 3 ms.

The number and chemical shifts of the resonances match the molecular structure and the fact that just one molecule is present in the asymetric unit. The sample that is recrystallized from xylene shows the same amount and position of the resonances as the one recrystallized from acetone (Figure S4). This strongly hints that the crystal structure and packing are the same. However, all resonances are broader, indicating less ordering in the sample.

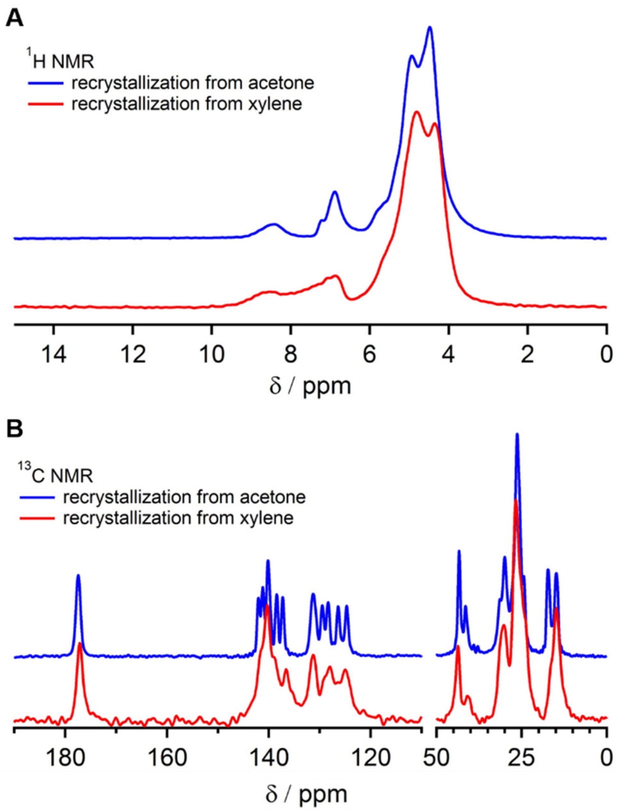

Figure S4. Solid-state NMR spectroscopy of powder samples of **3a**. ^{1}H **s**olid state NMRs (A) of **3a** prepared from acetone (blue) and xylene (red), respectively. ^{13}C **s**olid state NMRs (B) of **3a** prepared from acetone (blue) and xylene (red), respectively.

S6. FTIR spectroscopy of kinked bisamide 3a

Solid powder samples of kinked bisamide **3a** for FTIR spectroscopy were prepared from acetone and xylene as described in section S1 and S4. FTIR spectra were measured with a PerkinElmer 100 FTIR spectrometer equipped with an ATR sampling accessory. Measurements were performed in a wave number range of 650 cm^{-1} to 4000 cm^{-1} with a resolution of 4 cm^{-1}.

The FTIR spectra of **3a** from acetone (blue) and xylene (red) are shown in Figure S5 demonstrating that they are almost identical. Significant vibrations attributed to the amide groups are indicated by grey boxes. This includes the Amide A (N-H stretch vibrations) with a maximum of 3258 cm^{-1} (blue) and 3255 cm^{-1} (red), the Amide I (C=O stretch vibrations) both with a maximum of 1644 cm^{-1} and the Amide II (typically a superposition of C-N-H bend and C-N stretch vibrations) with a maximum at around 1515 cm^{-1} (blue) and 1514 cm^{-1} (red), respectively. We note that the N-H stretch vibrations is typically in the range of 3400 cm^{-1} for non-hydrogen bonded N-H groups. [8]

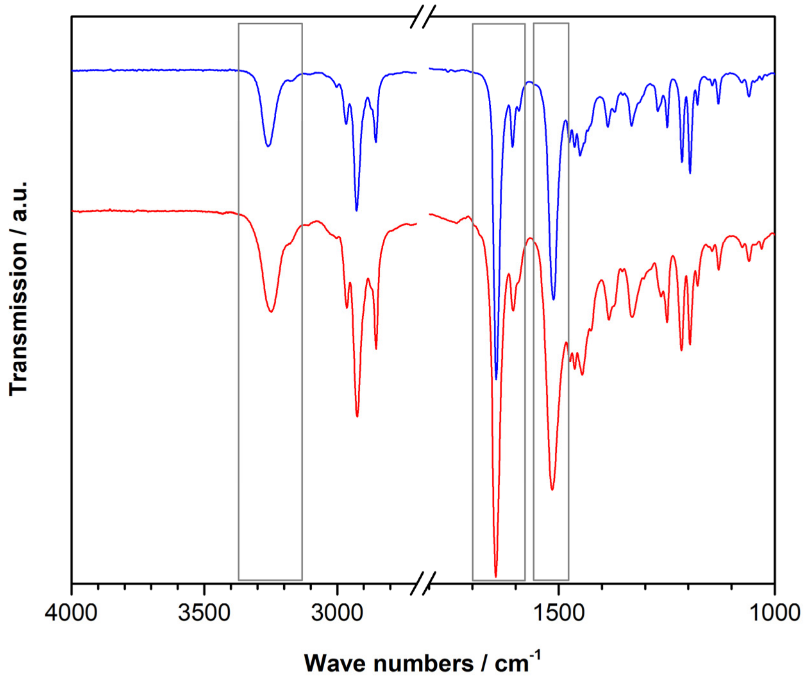

Figure S5. FT-IR spectra of **3a** prepared from acetone (blue) and xylene (red), respectively. The grey boxes indicate the Amide A (N-H stretch vibrations) with a maximum at around 3255 cm^{-1}, the Amide I (C=O stretch vibrations) with a maximum at around 1645 cm^{-1} and Amide II (superposition of N-H bend and C-N stretch vibrations) with a maximum at around 1515 cm^{-1}.

S7. Cell morphologies of polystyrene foams by batch foaming with kinked bisamides

Polystyrene foams with different supramolecular additve concentrations were prepared in a standard thermally-induced batch foam process as described in the experimental section comprising a master batch preparation, compounding and injection molding to polystyrene specimens, CO_2-saturation of the specimens at room temperature and foaming at elevated temperatures. Each foam was characterized and evaluated in view of morphology, cell size, cell density and foam density.

*Foam morphology and cell size histograms of PS batch foams with 1.0 wt.% of kinked bisamides **1a**, **2a** and **3a***

SEM micrographs of the cell morphologies and the respective histograms with the kinked bisamides **1a**, **2a** and **3a** are shown in Figure S6 revealing their influence at a concentration of 1.0 wt.%. While the PS foam containing 1.0 wt.% of 1a exhibits an inhomogeneous foam morphology with two types of cells and a mean cell size of 6.4 ± 3.5 µm, the PS foams with 1.0 wt.% of **2a** and **3a** show homogeneous cell morphologies with mean cell sizes of 3.5 ± 1.1 µm and 3.9 ± 1.3 µm, respectively. Moreover, the foam densities of the latter two are slightly lower with 52.1 ± 3.4 kg/m³ and 56.0 ± 3.5 kg/m³ compared to the foam with 1a with 69.8 ± 3.3 kg/m³.

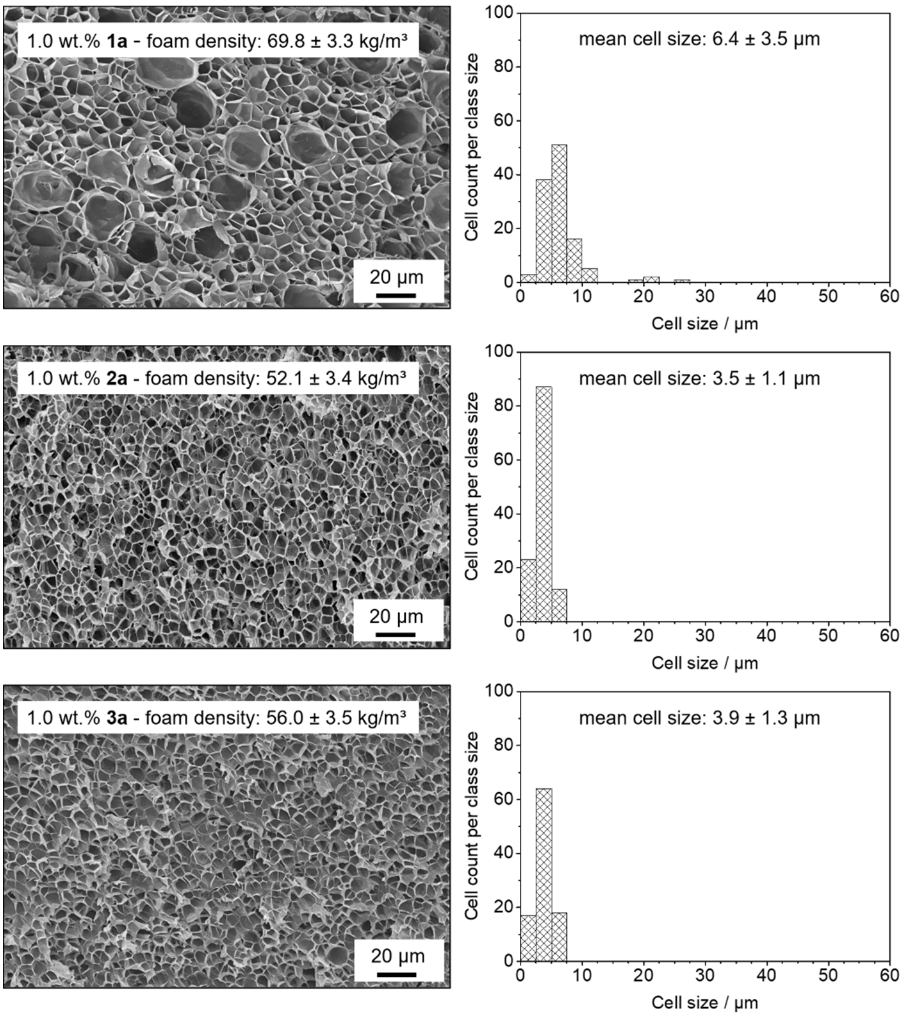

Figure S6. Left: SEM micrographs (500×) depicting the cell morphologies of batch foamed polystyrene specimens with 1.0 wt.% of kinked bisamides **1a**, **2a** and **3a**. The mean foam density is given for each concentration. Right: Corresponding histograms of the cell sizes including the mean cell sizes.

*Concentration-dependent evolution of the mean cell sizes of PS batch foams with kinked bisamides **1b**, **2b** and **3b***

Figure S7 shows the mean cell size of polystyrene batch foams with kinked bisamides with phenyl substituents **1b**, **2b** and **3b** at the concentrations of 1.0, 0.75, 0.5, 0.25, 0.1, 0.05, 0.025 and 0.01 wt.%. Exemplarily, the SEM micrographs for the concentration of 0.5 wt.% for each kinked bisamide are also shown. All kinked bisamides are capable of reducing the mean cell size in the investigated additive concentration range from 0.01 wt.% to 1.0 wt.% compared to the reference neat polystyrene foam. A significant reduction of the mean cell size by more than 40% was found at concentrations larger than 0.5 wt.%. Moreover, a significant reduction of the standard deviations of the mean cell sizes was achieved at these concentrations for the additives **1b**, **2b** and **3b**. Polystyrene foams with an additive concentration of 1.0 wt.% of **3b** was found to feature a microcellular morphology with the smallest foam cells (mean cell size of 5.8 ± 2.6 µm). The SEM micrographs at an additive concentration of 0.5 wt.% for all three batch foam samples (Figure S7) reveal foam morphologies with a pronounced homogeneity. All mean foam densities of the samples nucleated with **1b**, **2b** and **3b** are in the range between 53.7 – 82.9 kg/m^3. The mean foam density of the reference neat polystyrene was found to be 67.1 kg/m^3. Therefore, all foamed samples show low foam densities with volume expansion ratios between 10 – 20.

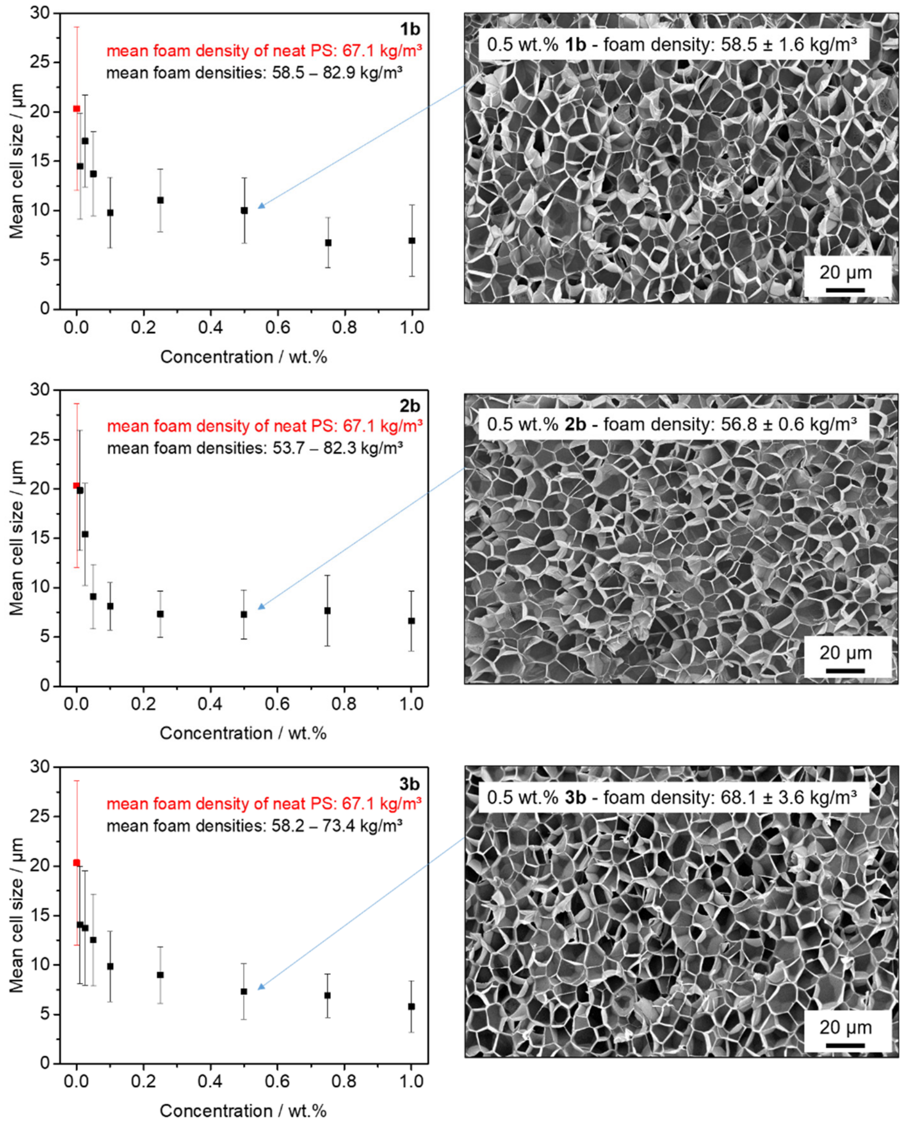

Figure S7. Left: Evolution of the mean cell size with increasing concentration of the kinked bisamides **1b, 2b** and **3b** of batch foamed polystyrene specimens. Data for the neat PS reference foam are shown in red. Right: SEM micrographs (500×) depicting the homogeneous microcellular morphology of batch foamed polystyrenes at a concentration of 0.5 wt.% of **1b, 2b** and **3b**, including the mean foam densities.

Concentration-dependent evolution of the mean cell sizes of PS batch foams with kinked bisamides ***1c****,* ***2c*** *and* ***3c***

In Figure S8, the mean cell size of polystyrene batch foams with kinked bisamides with tert-butyl substituents **1c**, **2c** and **3c** at the concentrations of 1.0, 0.75, 0.5, 0.25, 0.1, 0.05, 0.025 and 0.01 wt.% are depicted. Moreover, the SEM micrographs for the concentration of 0.5 wt.% for each kinked bisamide are shown. In a similar manner as described before, all kinked bisamides are capable of reducing the mean cell size in the investigated additive concentration range compared to the reference neat polystyrene sample. A significant but slightly smaller reduction of the mean cell size by more than 30% featuring also a reduction of the standard deviation was achieved at concentrations larger than 0.5 wt.%. PS foams with **2c** at an additive concentration of 1.0 wt.% yielded the smallest microcellular morphology with a mean cell size of 6.4 ± 1.9 µm. The SEM micrographs at an additive concentration of 0.5 wt.%

reveal homogeneous foam morphologies for all three batch foam samples. The mean foam densities of the samples nucleated with **1c** to **3c** are in the range between 51.6 – 124.4 kg/m³. Thus, most of the samples can be regarded as low-density polymer foams with volume expansion ratios between 9 – 20.

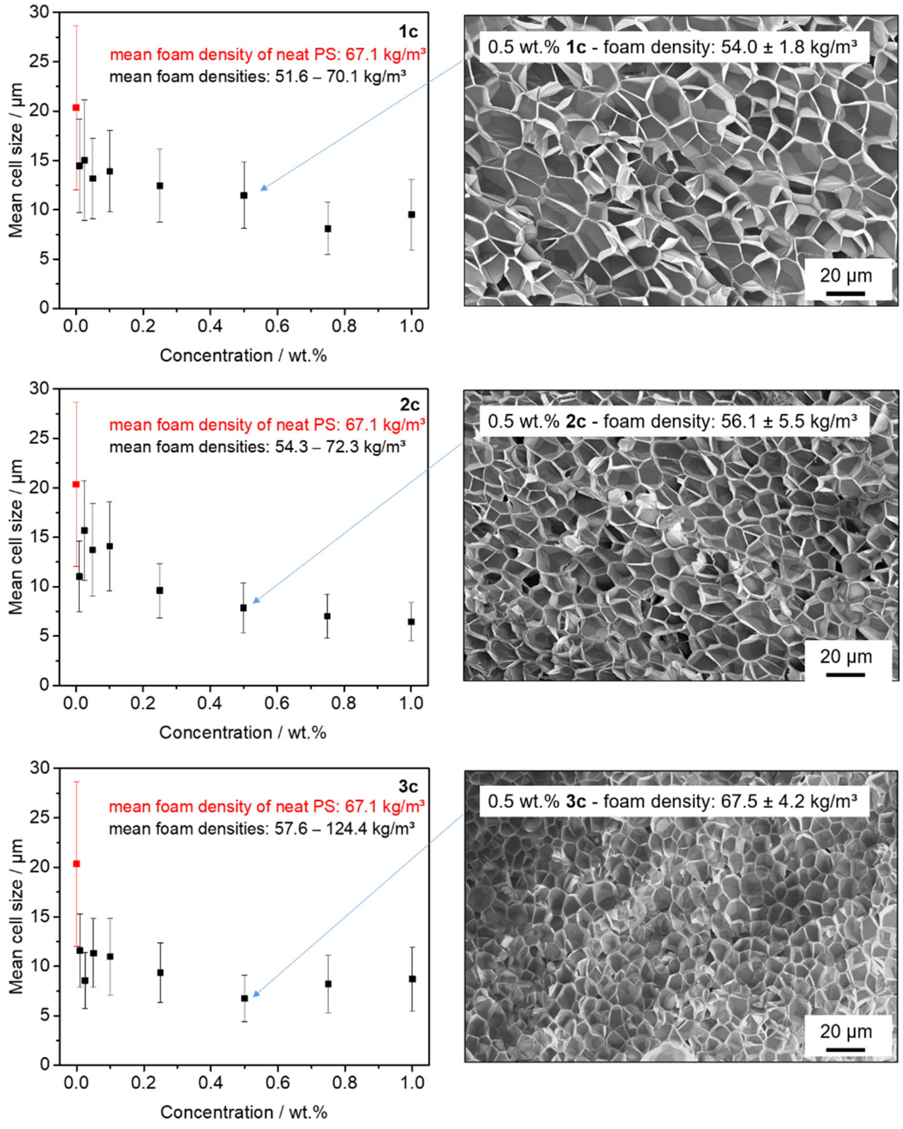

Figure S8. Left: Evolution of the mean cell size with increasing concentration of the kinked bisamides **1c, 2c** and **3c** of batch foamed polystyrene specimens. Data for the neat PS reference foam are shown in red. Right: SEM micrographs (500×) depicting the homogeneous microcellular morphology of batch foamed polystyrenes at a concentration of 0.5 wt.% of **1c, 2c** and **3c**, including the mean foam densities.

Concentration-dependent evolution of the mean cell sizes of PS batch foams with kinked bisamides ***1d*****,** ***2d*** *and* ***3d***

Figure S9 shows the mean cell size of polystyrene batch foams with kinked bisamides with n-butyl substituents **1d**, **2d** and **3d** at the concentrations of 1.0, 0.75, 0.5, 0.25, 0.1, 0.05, 0.025 and 0.01 wt.%. Exemplarily the SEM micrographs for the concentration of 0.5 wt.% for each bisamide is shown. All kinked bisamides are capable of reducing the mean cell size in the investigated additive concentration compared to the reference neat polystyrene sample. A significant reduction of the mean cell size by

more than 40% was found at concentrations larger than 0.5 wt.%. Moreover, a significant reduction of the standard deviations of the mean cell sizes was achieved at these concentrations. **2d** features the smallest microcellular morphology with a mean cell size of 5.2 ± 2.7 μm at an additive concentration of 0.75 wt.%. The SEM micrographs at an additive concentration of 0.5 wt.% reveal an inhomogeneous foam morphology for samples nucleated with **1d** and homogeneous foam morphologies for samples nucleated with **2d** and **3d**. The mean foam densities of the samples nucleated with **1d**, **2d** and **3d** are in the range between 49.2 – 76.0 kg/m³, while the mean foam density of the reference neat polystyrene is 67.1 kg/m³. Therefore, all foamed samples show low foam densities with volume expansion ratios between 15 – 20.

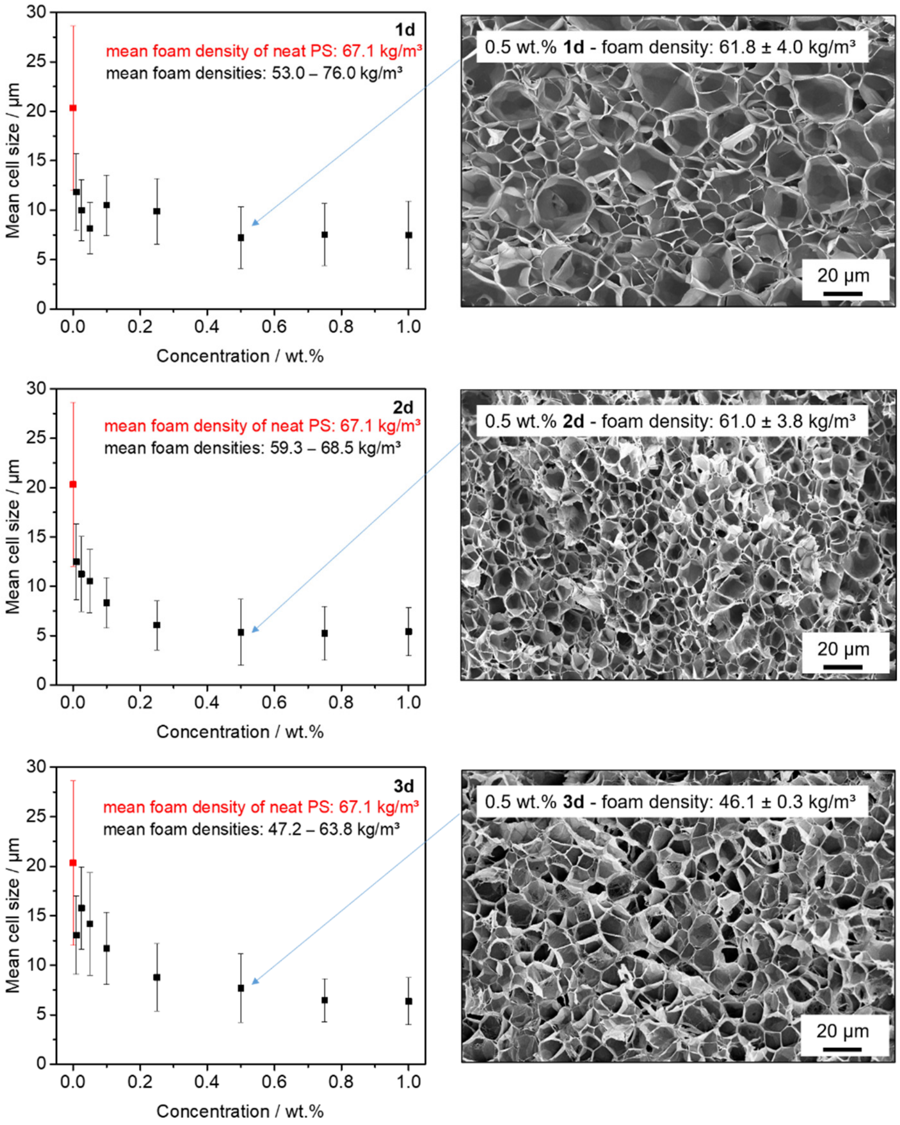

Figure S9. Left: Evolution of the mean cell size with increasing concentration of the kinked bisamides **1d, 2d** and **3d** of batch foamed polystyrene specimens. Data for the neat PS reference foam are shown in red. Right: SEM micrographs (500×) depicting the homogeneous microcellular morphology of batch foamed polystyrenes at a concentration of 0.5 wt.% of **1d**, **2d** and **3d**, including the mean foam densities.

S8. Summary of polystyrene batch foam properties with kinked bisamides

Foam densities and volume expansion ratio of polystyrene batch foams with kinked bisamides

Foam densities were calculated by the water-displacement method in agreement with ISO 1183 based on the Archimedes principle using a Mettler Toledo XP 205 with density kit. For this, a small rectangle was cut out of the specimen and weighed in air (m_{air}). Afterwards, the specimen was submerged in water and its apparent mass (m_{water}), which is reduced by the buoyant force, was measured. The resulting density (ρ_{foam}) was calculated with equation 1.

$$\rho_{foam} = \frac{m_{air}}{m_{air} - m_{water}} \cdot \rho_{water} \quad \text{with } \rho_{water}\ (296.3\ \text{K}) = 0.997541\ \text{g/cm}^3 \qquad (1)$$

Foam densities were measured on at least three cut samples from different locations of the respective foam and the mean values of the foam densities are given. All average foam densities are summarized in Table S3.

Table S3. Average foam densities of batch foamed polystyrene specimens with and without kinked bisamides.

**Foam density [kg/m³]**								
**neat PS**	67.1 ± 16.3							
	**Concentration of kinked bisamide [wt.%]**							
	**0.01**	**0.025**	**0.05**	**0.1**	**0.25**	**0.5**	**0.75**	**1.0**
**1a**	70.4 ± 2.9	65.4 ± 1.4	55.9 ± 1.6	72.8 ± 2.7	59.0 ± 2.3	50.0 ± 2.8	57.4 ± 5.7	69.8 ± 3.3
**2a**	58.2 ± 4.0	50.6 ± 0.6	59.9 ± 5.0	58.5 ± 2.7	49.2 ± 1.6	53.8 ± 3.0	50.3 ± 0.7	52.1 ± 3.4
**3a**	61.2 ± 2.9	47.8 ± 0.5	51.5 ± 5.3	54.5 ± 4.0	56.4 ± 5.4	46.8 ± 0.5	49.1 ± 3.7	56.0 ± 3.5
**1b**	82.9 ± 9.3	64.6 ± 1.4	71.4 ± 7.7	73.1 ± 1.6	61.4 ± 1.9	58.5 ± 1.6	63.2 ± 4.3	62.3 ± 3.7
**2b**	61.5 ± 1.0	56.8 ± 1.1	58.9 ± 5.7	76.7 ± 1.8	74.8 ± 2.2	56.8 ± 0.6	82.3 ± 0.5	53.7 ± 1.2
**3b**	71.6 ± 3.6	63.6 ± 1.6	58.2 ± 4.5	71.2 ± 8.9	73.4 ± 2.7	68.1 ± 3.6	65.7 ± 10.3	62.5 ± 0.8
**1c**	53.7 ± 2.5	63.6 ± 6.4	70.1 ± 5.8	58.2 ± 1.4	55.7 ± 6.2	54.0 ± 1.8	62.5 ± 3.5	51.6 ± 3.5
**2c**	72.3 ± 2.1	56.8 ± 3.6	67.9 ± 3.6	61.2 ± 2.9	63.8 ± 9.5	56.1 ± 5.5	54.3 ± 2.3	66.2 ± 5.4
**3c**	124.4 ± 9.4	77.9 ± 2.7	59.4 ± 1.6	61.5 ± 4.1	64.3 ± 5.7	67.5 ± 4.2	57.6 ± 4.6	57.6 ± 2.6
**1d**	66.9 ± 2.6	62.3 ± 4.5	74.9 ± 10.0	76.0 ± 3.4	62.7 ± 1.6	61.8 ± 4.0	53.0 ± 1.9	61.8 ± 4.3
**2d**	68.5 ± 2.6	59.3 ± 5.1	64.7 ± 5.7	59.5 ± 2.4	59.6 ± 1.4	61.0 ± 3.8	65.5 ± 5.8	65.6 ± 5.1
**3d**	63.8 ± 3.9	47.2 ± 0.3	60.4 ± 7.1	53.0 ± 0.9	50.1 ± 0.8	46.1 ± 0.3	47.4 ± 1.6	51.2 ± 3.0

The volume expansion ratios (VER) were calculated by dividing the average foam densities by the polystyrene density, according to equation 2.

$$VER = \frac{\rho_{PS,bulk}}{\rho_{foam}} \quad \text{with } \rho_{PS,bulk} = 1040 \text{ kg/m}^3 \qquad (2)$$

All VER are summarized in Table S4.

Table S4. Volume expansion ratio of batch foamed polystyrene specimens with and without kinked bisamides.

**VER**								
**neat PS**	15.5							
	**Concentration of kinked bisamide [wt.%]**							
	**0.01**	**0.025**	**0.05**	**0.1**	**0.25**	**0.5**	**0.75**	**1.0**
**1a**	14.8	15.9	18.6	14.3	17.6	20.8	18.1	14.9
**2a**	17.9	20.6	17.4	17.8	21.1	19.3	20.7	20.0
**3a**	17.0	21.8	20.2	19.1	18.4	22.2	21.2	18.6
**1b**	12.5	16.1	14.6	14.2	16.9	17.8	16.5	16.7
**2b**	16.9	18.3	17.7	13.6	13.9	18.3	12.6	19.4
**3b**	14.5	16.4	17.9	14.6	14.2	15.3	15.8	16.6
**1c**	19.4	16.4	14.8	17.9	18.7	19.3	16.6	20.2
**2c**	14.4	18.3	15.3	17.0	16.3	18.5	19.2	15.7
**3c**	8.4	13.4	17.5	16.9	16.2	15.4	18.1	18.1
**1d**	15.5	16.7	13.9	13.7	16.6	16.8	19.6	16.8
**2d**	15.2	17.5	16.1	17.5	17.4	17.0	15.9	15.9
**3d**	16.3	22.0	17.2	19.6	20.8	22.6	21.9	20.3

Cell densities of polystyrene batch foams with kinked bisamides

Cell densities ρ_{cell} with respect to the unfoamed solid polymer were calculated according to equation 3, with N_c the number of cells in the selected area, A_S the area of the selected section and the volume expansion ratio. The results of the cell densities are summarized in Table S5.

$$\rho_{cell} = \left(\frac{N_c}{A_S}\right)^{\frac{3}{2}} \cdot VER \quad (3)$$

Table S5. Cell densities of batch foamed polystyrene specimens with and without kinked bisamides.

**Cell density [cm^{-3}]**								
**neat PS**	2.14E9							
	**Concentration of kinked bisamide [wt.%]**							
	**0.01**	**0.025**	**0.05**	**0.1**	**0.25**	**0.5**	**0.75**	**1.0**
**1a**	1.25E10	9.80E09	1.58E10	3.13E10	3.88E10	4.16E10	5.85E10	5.02E10
**2a**	8.45E09	7.44E09	8.99E09	9.71E09	7.17E10	4.64E11	2.79E11	5.67E11
**3a**	8.79E09	8.88E09	6.44E09	2.08E10	7.49E10	1.89E11	3.05E11	3.49E11
**1b**	6.25E09	5.72E09	7.94E09	2.22E10	2.08E10	2.83E10	7.59E10	5.79E10
**2b**	3.15E09	6.74E09	2.90E10	3.09E10	4.52E10	5.40E10	3.12E10	3.47E10
**3b**	9.79E09	8.91E09	1.25E10	2.19E10	3.19E10	5.24E10	2.53E10	9.37E10
**1c**	9.78E09	7.34E09	8.47E09	1.06E10	1.61E10	1.91E10	4.69E10	3.16E10
**2c**	1.48E10	6.74E09	7.28E09	7.49E09	2.58E10	4.34E10	5.32E10	7.02E10
**3c**	7.06E09	3.15E10	1.67E10	1.68E10	2.83E10	5.89E10	4.57E10	3.65E10
**1d**	1.29E10	2.27E10	3.60E10	1.98E10	2.17E10	4.31E10	5.46E10	4.70E10
**2d**	1.11E10	1.65E10	2.09E10	4.04E10	8.79E10	1.10E11	1.13E11	1.10E11
**3d**	9.00E09	7.69E09	8.01E09	2.20E10	3.90E10	6.38E10	9.04E10	8.59E10

S9. Photograph of an extruded neat polystyrene foam and a polystyrene foam with 0.5 wt.% 3a

Foam extrusion experiments were carried out using a tandem extrusion line (Dr. Collin GmbH) comprising a twin-screw extruder with a 25 mm screw and a L/D ratio of 42 (A-Extruder) and a single-screw extruder with a 45 mm screw and L/D ratio of 30 (B-Extruder) equipped with a slit die with a gap of 0.6 mm and a width of 30 mm. For the extrusion foaming with kinked bisamide **3a**, a 5.0 wt.% masterbatch powder-powder mixture was used and foams were prepraed by diluting the masterbatch with neat PS granulates. 4 wt.% CO_2 and 3 wt.% EtOH was used as physical blowing agent. As processing parameters, a screw speed of 8 rpm at the B-Extruder with an overall throughput of 4.5 kg h^{-1} was selected. The melt temperature in the A-Extruder was adjusted to 220 °C. The melt temperature near to the outlet of the B-extruder and the die temperature were selected between 110 – 120 °C and 126 °C, respectively. The PS reference foam was prepared using neat PS granulates in the same manner. Figure S10 shows exemplarily photographs of non-calibrated extruded foams of a neat polystyrene reference foam and polystyrene foam with 0.5 wt.% **3a** featuring almost the same foam density but very different cell sizes.

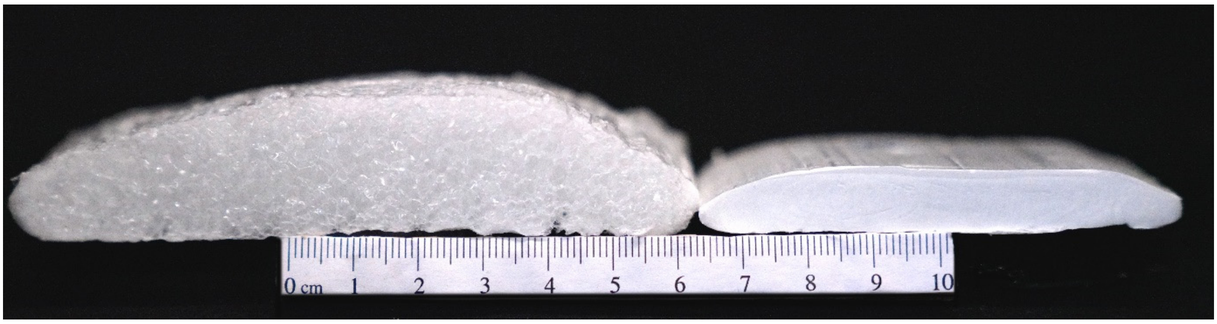

Figure S10. Photograph of a neat extruded polystyrene foam (left) with a mean foam density of 61.6 ± 4.1 kg/m³ and a mean cell size of 1094 ± 377 µm and an extruded polystyrene foam with 0.5 wt.% **3a** (right) with a mean foam density of 63.9 ± 1.9 kg/m³ and a mean cell size of 18.8 ± 8.5 µm, produced at very similar processing conditions.

S10. Photograph and light microscopy image of neat polystyrene foams by extrusion foaming

Extruded foams of neat polystyrene foam possess very large cell sizes, which cannot reliably be determined by means of scanning electron microscopy. Thus, photographs and light microscopy images were taken (Figure S11) and evaluated. For this, a small rectangle was cut out of the neat polystyrene foam. The interface was colored black to make the cell morphology visible. Figure S11 A shows a photograph of an inhomogeneous cell morphology with cell sizes in the range between 0.1 mm and 2.0 mm. Figure S11 B shows a micrograph of a foam cell demonstrating that neat polystyrene foams feature comparable thick cell walls in the range between 50 μm and 150 μm.

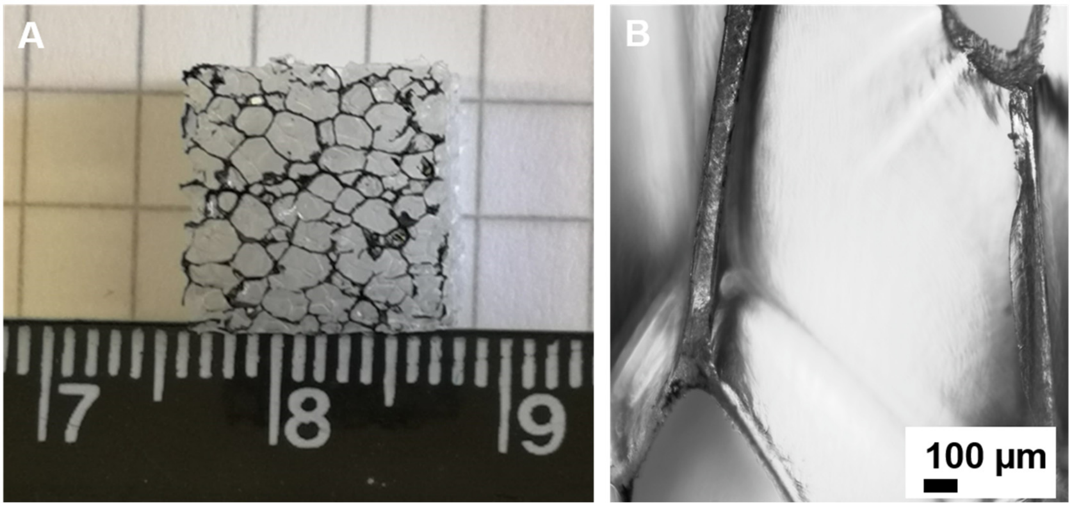

Figure S11. Photograph (A) and light microscopy image (B,10×) of extruded foams of neat polystyrene.

References

1. Clark, S.J.; Segall, M.D.; Pickard, C.J.; Hasnip, P.J.; Probert, M.I.J.; Refson, K.; Payne, M.C. First principles methods using CASTEP. *Z. Kristallogr.* **2005**, *220*, 567–570.
2. Coelho, A.A. TOPAS and TOPAS-Academic : an optimization program integrating computer algebra and crystallographic objects written in C++. *J Appl Crystallogr* **2018**, *51*, 210–218.
3. Putz, H.; Schön, J.C.; Jansen, M. Combined method for ab initio structure solution from powder diffraction data. *J Appl Crystallogr* **1999**, *32*, 864–870.
4. Järvinen, M. Application of symmetrized harmonics expansion to correction of the preferred orientation effect. *J Appl Crystallogr* **1993**, *26*, 525–531.
5. Sakellariou, D.; Lesage, A.; Hodgkinson, P.; Emsley, L. Homonuclear dipolar decoupling in solid-state NMR using continuous phase modulation. *Chem. Phys. Lett.* **2000**, *319*, 253–260.
6. Metz, G.; Wu, X.L.; Smith, S.O. Ramped-Amplitude Cross Polarization in Magic-Angle-Spinning NMR. *J Magn Reson. Series A* **1994**, *110*, 219–227.
7. Fung, B.M.; Khitrin, A.K.; Ermolaev, K. An improved broadband decoupling sequence for liquid crystals and solids. *J Magn Reson.* **2000**, *142*, 97–101.
8. Günzler, H.; Gremlich, H.-U. *IR-Spektroskopie;* Wiley-VCH Verlag GmbH & Co. KGaA: Weinheim, Germany, 2003, ISBN 9783527662852.
